# Supplementary material for: Validation of revised patient measures of safety: PMOS-30 and PMOS-10
Source: BMJ Open. 2019 Nov 28;9(11):e031355. doi: 10.1136/bmjopen-2019-031355 (PMC6924707; doi:10.1136/bmjopen-2019-031355)
Supplement: Author's manuscript [file bmjopen-2019-031355.draft_revisions.pdf]

BMJ Open

BMJ Open is committed to open peer review. As part of this commitment we make the peer review history of every article we publish publicly available.

When an article is published we post the peer reviewers' comments and the authors' responses online. We also post the versions of the paper that were used during peer review. These are the versions that the peer review comments apply to.

The versions of the paper that follow are the versions that were submitted during the peer review process. They are not the versions of record or the final published versions. They should not be cited or distributed as the published version of this manuscript.

BMJ Open is an open access journal and the full, final, typeset and author-corrected version of record of the manuscript is available on our site with no access controls, subscription charges or pay-per-view fees (http://bmjopen.bmj.com).

If you have any questions on BMJ Open's open peer review process please email info.bmjopen@bmj.com

BMJ Open

Validation of revised Patient Measures of Safety: PMOS-30 and PMOS-10

Journal:	*BMJ Open*
Manuscript ID	bmjopen-2019-031355
Article Type:	Research
Date Submitted by the Author:	30-Apr-2019

Complete List of Authors:	Louch, Gemma; Bradford Institute for Health Research, Yorkshire Quality and Safety Research Group<br>Reynolds , Caroline ; Bradford Institute for Health Research, Yorkshire Quality and Safety Research Group<br>Moore, Sally; Bradford Institute for Health Research, Yorkshire Quality and Safety Research Group<br>Marsh, Claire; Bradford Institute for Health Research, Yorkshire Quality and Safety Research Group<br>Heyhoe, Jane; Bradford Institute for Health Research, Yorkshire Quality and Safety Research Group<br>Albutt, Abigail; Bradford Institute for Health Research, Yorkshire Quality and Safety Research Group<br>Lawton, Rebecca; University of Leeds, School of Psychology; Bradford Institute for Health Research, Yorkshire Quality and Safety Research Group

Keywords:	patient safety, patient involvement, psychometrics, hospitals, feedback

SCHOLARONE™
Manuscripts

Validation of revised Patient Measures of Safety: PMOS-30 and PMOS-10

Gemma Louch

Yorkshire Quality and Safety Research Group, Bradford Institute for Health Research, Bradford Royal Infirmary, Bradford, BD9 6RJ, UK

Caroline Reynolds

Yorkshire Quality and Safety Research Group, Bradford Institute for Health Research, Bradford Royal Infirmary, Bradford, BD9 6RJ, UK

Sally Moore

Yorkshire Quality and Safety Research Group, Bradford Institute for Health Research, Bradford Royal Infirmary, Bradford, BD9 6RJ, UK

Claire Marsh

Yorkshire Quality and Safety Research Group, Bradford Institute for Health Research, Bradford Royal Infirmary, Bradford, BD9 6RJ, UK

Jane Heyhoe

Yorkshire Quality and Safety Research Group, Bradford Institute for Health Research, Bradford Royal Infirmary, Bradford, BD9 6RJ, UK

Abigail Albutt

Yorkshire Quality and Safety Research Group, Bradford Institute for Health Research, Bradford Royal Infirmary, Bradford, BD9 6RJ, UK

Rebecca Lawton

Yorkshire Quality and Safety Research Group, Bradford Institute for Health Research, Bradford Royal Infirmary, Bradford, BD9 6RJ, UK and
School of Psychology, University of Leeds, Leeds, LS2 9JT, UK

and on behalf of the Yorkshire Quality and Safety Research Group

Correspondence

Gemma Louch: Gemma.Louch@bthft.nhs.uk

01274 383413

Yorkshire Quality and Safety Research Group, Bradford Institute for Health Research, Bradford Royal Infirmary, Bradford, BD9 6RJ, UK

Word count: 3981 (excluding title page, abstract, references and tables).

Keywords: feedback, patient safety, patient involvement, hospitals, psychometrics

For peer review only - http://bmjopen.bmj.com/site/about/guidelines.xhtml

Abstract

Objectives: There is growing evidence that patients can provide feedback on the safety of their care. The 44-item Patient Measure of Safety (PMOS) was developed for this purpose. While valid and reliable, the length of this questionnaire makes it potentially challenging for routine use. Our study aimed to produce revised, shortened versions of PMOS (PMOS-30 and PMOS-10), which retained the psychometric properties of the longer version.

Participants: In phase 1 to produce a shortened 'diagnostic' measure we analysed data from 2002 patients who completed PMOS-44, and examined the reliability of the revised measure (PMOS-30) in a sample of 425 patients. In phase 2, to produce a brief standalone measure, we again analysed data from 2002 patients who completed PMOS-44, and tested the reliability and validity of the brief standalone measure (PMOS-10) in a sample of 165 patients.

Methods: Phase 1 involved a combination of secondary data analysis (e.g. standard deviation and inter-item correlations) and a consensus group exercise to produce PMOS-30 and examine face validity. Secondary analysis of PMOS-30 data examined reliability (e.g. Cronbach's alpha). Phase 2 involved secondary data analysis (e.g. corrected item-total correlations) to produce PMOS-10, and primary data collection to assess its reliability and validity (e.g. Cronbach's alpha, analysis of variance).

Results: Fourteen items were removed to produce PMOS-30 and the percentage of negatively worded items was reduced from 57% to 33%. PMOS-30 demonstrated good internal reliability. The 10 items with the highest corrected item-total correlations across both PMOS-44 and PMOS-30 composed PMOS-10. PMOS-10 had good internal reliabilty, demonstrated convergent validity, however, discriminant validity was not established.

Conclusions: Two revised, shortened versions of the original PMOS-44 (PMOS-30 and PMOS-10) were produced to capture patient feedback about safety in hospital. The measures demonstrated good reliability and validity, and preserved the psychometric properties of the original measure.

For peer review only - http://bmjopen.bmj.com/site/about/guidelines.xhtml

Strengths and limitations of this study

- Large sample size of 2002 patient participants used in the stage 1 initial PMOS-30 shortening exercise
- Reliability testing of PMOS-30 took place in a variety of wards across three hospital trusts
- The involvement of patient representatives is a key strength of the PMOS-10 development phase
- A limitation of our work is that our studies only included patient participants who could understand English

For peer review only

Background

Measurement has posed an ongoing challenge in patient safety improvement. The difficulties and tensions associated with developing and implementing measures of patient safety are well recognised, and there is a need for measures to be relevant to multiple stakeholders, have a scientific basis, as well as being feasible and usable.[1] Indeed, there have been calls for more time and money to be invested in the science of safety measurement and the implementation of systems to capture such measures in order to understand whether safety is improving.[2]

In a 2013 report, Vincent and colleagues proposed The Measurement and Monitoring of Safety Framework (MMSF).[3] The framework focusses on moving beyond the measurement of past harm, to the prevention of future harm across five dimensions of safety measurement and monitoring: 1) Past harm; 2 Reliability; 3) Sensitivity to operations; 4) Anticipation and preparedness; and 5) Integration and learning. Crucially, the report emphasised that safety cannot be captured in a single measure, and recognised that patients and carers play an essential role in safety monitoring, but are often an underused resource.

The collection of feedback on patient satisfaction and experiences of care is now commonplace,[4] and in the UK, multiple high profile reports have highlighted the importance of listening and responding to patient views about safety.[5-7] There is growing evidence that patients can provide feedback on the safety of their care,[8, 9] and research to suggest that patients can offer a unique perspective on patient safety not captured by other established methods of incident detection.[10]

To provide a proactive diagnostic tool which systematically invites the patient perspective on safety, researchers from the UK developed an intervention to provide a theory and evidence-

For peer review only - http://bmjopen.bmj.com/site/about/guidelines.xhtml

based approach for the collection of hospital in-patient feedback about safety to support service improvement. The Patient Reporting and Action for a Safe Environment (PRASE) intervention[11, 12] includes two measurement tools. The first is a 44-item theory based measure—the Patient Measure of Safety (PMOS),[13, 14] informed by the domains of the Yorkshire Contributory Factors Framework (YCFF).[15] The second is the Patient Incident Reporting Tool (PIRT)[12] which enables patients to report safety concerns and positive experiences. The principal idea of the PRASE intervention is that feedback on the safety of care is collected from patients, and this feedback can be used at the ward level to support service improvement on a routine basis.

The reliability and validity of the original PMOS has been established previously.[13, 14] The original measure included a minimum of two items per domain, across nine domains, known to contribute to safety incidents in hospitals: (1) Communication and teamworking; (2) Organisation and care planning; (3) Access to resources; (4) Ward type and layout; (5) Information flow; (6) Staff roles and responsibilities; (7) Staff training; (8) Equipment (design and functioning); (9) Delays. Within a Randomised Controlled Trial (RCT) of the PRASE intervention,[11] PMOS data was collected from over two thousand patient participants. The fieldworkers who facilitated this data collection reported that some patients, particularly those who were older or very unwell, struggled with the length of the measure and the phrasing of some of the negatively worded items. Evidence suggests that shorter measures and a reduction in assessment time can lessen the burden on participants and improve response rates and missing data.[16] Therefore, for a large number of questionnaires used in a healthcare context, often short form or brief measures are produced to address this issue with the aim of maintaining acceptable reliability and validity (e.g.[17, 18]).

Whilst in the PRASE RCT[11] researchers were on hand to answer questions and encourage responses, so the above issues did not pose a major problem (response rates were 86%), these issues may result in lower uptake as use becomes more widespread. For example, if data are collected via hospital volunteers (one option that has been tested), difficulties with question wording and/or the length of the measure pose a threat to the reliability and validity of the responses, and may result in missing data. Indeed, in a formative evaluation of the implementation of PRASE with hospital volunteers—volunteers described how the measure was too time consuming for patients to complete, and that its length impacted on the conversation and rapport they were able to build with patients.[19] Furthermore, in recognition of the potential difficulties described above, researchers in Australia have produced a revised version of the measure for use with vulnerable older adult groups.[20]

For peer review only - http://bmjopen.bmj.com/site/about/guidelines.xhtml

As it is currently conceived, the PMOS has a diagnostic function. It can be used to identify those factors (see domains above) where attention might most usefully be focused when planning improvement. The first aim therefore was to produce a revised version of PMOS that was shorter with improved acceptability, whilst at the same time preserving the psychometric properties, conceptual underpinnings and diagnostic function of the longer version. This version would continue to be used, alongside the PIRT tool as a basis for the PRASE intervention (Phase 1). Our second aim was to develop a version of PMOS that was much shorter and that could be used as a brief standalone measure for on-going monitoring of ward safety performance not focussed on planning improvement. Potentially this measure could be used to prompt additional information gathering effort using the refined diagnostic measure (Phase 2) or to monitor safety performance over time.

Objectives

The objectives of this research were to produce two measures that are practical, feasible and simple for patients to complete.

Phase 1 focussed on the diagnostic measure and aimed to reduce the number of PMOS items, rephrase items where appropriate, consider face and content validity (stage 1), and establish the internal reliability of the revised diagnostic measure (stage 2).

Phase 2 focussed on producing a brief standalone measure for on-going monitoring of ward safety performance (stage 1) and establishing its internal reliability, face validity, convergent validity and discriminant validity (stage 2).

Patient and public involvement statement

Patients and the public were involved in this study, specifically the Yorkshire Quality and Safety Research (YQSR) Group patient panel and hospital volunteers from Bradford Teaching Hospitals NHS Foundation Trust. More information is provided in the phase 1 and phase 2 methods sections respectively.

For peer review only

Phase 1

Method

Participants

Stage 1: Shortening exercise to produce revised diagnostic measure

The shortening exercise included the analysis of PMOS data collected using the original measure (44 items) within the RCT of the PRASE intervention.[11] The analysis included 2002 patient participants who were recruited across three hospital trusts and 33 wards in the UK between May 2013 and September 2014. This exercise also included an iterative consensus approach in a series of meetings with researchers from the Yorkshire Quality and Safety

For peer review only - http://bmjopen.bmj.com/site/about/guidelines.xhtml

Research (YQSR) Group who developed the original measure, and researchers with experience of facilitating the collection of PMOS data in the RCT of the PRASE intervention. These meetings aimed to reduce the number of items in the measure, and considered the phrasing of retained items. At this stage, to establish face validity, we also sought input on item wordings/phrasings from two patient panel representatives (from the YQSR Group's patient panel), and two hospital volunteers involved in a project implementing PRASE in collaboration with hospital volunteers, which we henceforth refer to as the hospital volunteers project[a].[19, 21]

Stage 2: Revised diagnostic measure reliability

Subsequently, the internal reliability of the revised PMOS was tested in a sample of 425 patients recruited by patient volunteers across 17 wards from three hospitals, at three hospital trusts in the UK between September 2015 and May 2016, within the hospital volunteers project. The characteristics of the patient samples are presented in Table 1.

Table 1. Characteristics of the patient samples

	**PMOS-44**<br>**N = 2002**		**PMOS-30**<br>**N = 425**	
**Age mean (SD)**	62.48 (17.28)		52.38 (26.34)	
**Gender**	Female = 50.0%<br>Male = 50.0%		Female = 48.7%<br>Male = 49.2%	
**Length of stay in days mean (SD)**	6.94 (12.47)		6.35 (7.71)	
**Ethnicity**	African<br>Bangladeshi<br>British<br>Caribbean<br>Chinese<br>Indian<br>Irish<br>Other<br>Other Background<br>Other Ethnic Background<br>Other Mixed Background<br>Pakistani<br>White & Black Caribbean	.3%<br>.1%<br>94.5%<br>.6%<br>.2%<br>.5%<br>.6%<br>1.3%<br>.4%<br>.1%<br>.2%<br>1.1%<br>.3%	African<br>Bangladeshi<br>British<br>Indian<br>Irish<br>Other<br>Other Ethnic Background<br>Pakistani<br>White & Asian	.2%<br>.2%<br>90.6%<br>1.6%<br>.5%<br>1.6%<br>.5%<br>4.2%<br>.2%

Note. SD = standard deviation.

Analysis

All study 1 and study 2 analyses were performed using SPSS Version 23.[22] PMOS responses were recoded such that higher scores indicated a more favourable perception of safety. Our analytical approach was informed by the original PMOS validation.[14]

a https://www.health.org.uk/improvement-projects/putting-the-patient-at-the-heart-of-patient-safety.

For peer review only - http://bmjopen.bmj.com/site/about/guidelines.xhtml

For peer review only

Stage 1: Shortening exercise for the diagnostic measure

Fieldworkers who collected PMOS data from patients within the RCT of the PRASE intervention met to discuss the content of the original measure. Potential items for removal were highlighted, for example, based on patient difficulties in understanding of the item or patient feedback that items were repetitive. Analyses were conducted to examine item response variation (standard deviation) and inter-item correlations[b] using Pearson's correlation. Research suggests that for a 5-point scale items which demonstrate reasonable variability (standard deviation of 1.00 or higher) are potential items to retain and that items with limited variance are candidates for removal.[23] On this basis, items demonstrating low variability (i.e. <.7) were identified as candidate items for removal, and in terms of the level of overlap between items (possible multicollinearity), high inter-item correlations highlighted candidate items for removal, i.e. higher than .5[c].[24] Subsequently, in a series of consensus meetings with the wider research group, items which had been identified as problematic, based on either fieldworker perceptions or the item analyses, were discussed in greater detail. For the purpose of construct validity, the group considered each potential item for removal, and focussed on whether each item reliably tapped what it was intended to measure. For the purpose of content validity we also endeavoured to ensure a minimum of two items per domain were retained. We used multiple criteria, rather than only a statistical approach when considering items for removal to safeguard against key items being removed.

Stage 2: Revised diagnostic measure reliability

Internal reliability

We examined the internal consistency reliability[d] (Cronbach's alpha) of PMOS-30 data collected from 425 hospital in-patients within the hospital volunteers project. In line with guidance, a reliability coefficient α of >.7 was deemed acceptable.[25, 26] For domains with three or more items we examined the internal consistency reliability in terms of original measure's domain structure i.e. the domains that form the basis of the feedback to support service improvement in the PRASE intervention, and assessed the average inter-item correlations[e] using the recommended values range of .15 to .50.[27] For domains with three or more items we assessed inter-item correlations.

---

b Missing data were excluded list-wise.
c There were no inter-item correlations >.5.
d Missing data were excluded list-wise.
e Missing data were excluded list-wise.

For peer review only - http://bmjopen.bmj.com/site/about/guidelines.xhtml

Results

Stage 1: Shortening exercise for the diagnostic measure

Descriptive statistics

Of the 44 items in the original measure, 14 items were removed (32%). Table 2 presents the rationale for the removal of each item. Eight items were removed due to both statistical reasons and fieldworker feedback, five items were removed due to fieldworker feedback alone, and one item was removed due to statistical reasons alone. To facilitate the likelihood of reliable and valid responses, we also reduced the quantity of negatively phrased items in the revised measure. In the original measure the proportion of negatively phrased items was 57% (25 items); in the revised measure this was reduced to 33% (10 items). See Table 3 for the rephrasing of retained items.[f]

For peer review only

f The revised 30-item measure (PMOS-30) and an 'easy read' version of the measure which has been subsequently produced - can be requested via https://www.improvementacademy.org/tools-and-resources/patient-reporting-and-action-for-a-safe-environment.html.

For peer review only - http://bmjopen.bmj.com/site/about/guidelines.xhtml

Table 2. Rationale for item removal

Item removed	Rationale	
	Consensus group discussion	***Standard deviation***
The following aspects of the ward made it difficult for staff to do their jobs: Lighting levels	Fieldworkers highlighted that frequently patients did not respond to this item with regards to themselves, and therefore the item was often not answered as intended i.e. how lighting levels affect staff	Low variability SD = .56
Staff were always able to get advice from other teams about my care if needed	Fieldworkers highlighted that frequently patients did not respond to this item in terms of what the item is attempting to tap i.e. specialist staff etc.	Low variability SD = .67
When staff talked about my care with others the information they shared was correct	Fieldworkers highlighted that frequently patients did not respond to this item in terms of what the item is attempting to tap i.e. information sharing between staff	Low variability SD = .60
The following aspects of the ward made it difficult for staff to do their jobs: Position of nurses' station	Fieldworkers highlighted that participants often struggled to respond to this item from the patient perspective	
There was equipment that staff found difficult to use (e.g. monitoring equipment, beds, hoists)	Fieldworkers highlighted that frequently patients reported that it would be more appropriate for staff to respond to this item	Low variability SD = .65
Staff were kept waiting for my test results	Fieldworkers felt this issue was covered elsewhere in the measure i.e. 'Information about me that my health care team needed was always available'	
The following aspects of the ward made it uncomfortable for me: Lack of Space	Fieldworkers felt that lack of space was assessed elsewhere in the measure, albeit in relation to staff rather than patients. 'The following aspects of the ward made it difficult for staff to do their jobs: Lack of space'	
Staff always seemed to know what they were meant to be doing	Fieldworkers highlighted that patients often felt they could not answer this. Fieldworkers also felt there was conceptual overlap with another item i.e. 'I got answers to all the questions I had about my care'	Low variability SD = .59

For peer review only - http://bmjopen.bmj.com/site/about/guidelines.xhtml

Item removed	Rationale	
	Consensus group discussion	***Standard deviation***
Staff always agreed about my treatment/care	Fieldworkers felt there was conceptual overlap with another item i.e. 'Staff gave me different information about my care'	Low variability SD = .61
Nurses were always able to get help from other staff when they asked for it	Fieldworkers felt there was conceptual overlap with another item i.e. 'Staff seemed to struggle to get help when they needed it'	Low variability SD = .57
I knew who to go to if I needed to ask a question	Fieldworkers felt other items capture this issue	
The following aspects of the ward made it difficult for staff to do their jobs: Clutter & untidiness		Low variability SD = .67
Equipment needed for my care was always working properly	Fieldworkers highlighted that frequently patients reported that it would be more appropriate for staff to respond to this item	Low variability SD = .71
The following aspects of the ward made it uncomfortable for me: Other - Please specify	Fieldworkers felt this item could be removed due to low response rate	

Note. SD = standard deviation.

For peer review only - http://bmjopen.bmj.com/site/about/guidelines.xhtml

Table 3. Retained items rephrasing

**PMOS-44 item**	**PMOS-30 item**
Sometimes there was no-one available to deal with aspects of my care	There was always someone available to deal with every aspect of my care
Staff did not work together as a team here	Staff worked together as a team here
The drugs I have been prescribed were always available in the hospital	My medicines were always available
A doctor changed my plan of care and other staff didn't know about it	Staff didn't always know when a doctor changed my plan of care
Staff gave me different information about my care	Staff gave me conflicting information about my care
I have needed treatment and there was no-one available who was trained to do it	When I needed treatment there was always someone available who was trained to do it
Equipment and supplies were not always available when needed (e.g. hoists, bed pans, drugs)	Equipment and supplies were always available when needed (e.g. hoists, bed pans, walking aids, dressings)
I was on a ward that was not able to deal with my treatment needs	The ward was able to deal with all my treatment needs
After a shift change staff did not appear to know important information about my care	After shift changes staff knew important information about my care
It was clear who was in charge of the staff	It was clear who was in charge of the ward staff
I always knew which nurse was responsible for my care	I always knew which nurse or nurses were responsible for my care
On at least one occasion a member of staff was not able to use the necessary equipment	Staff were always able to use the necessary equipment
On at least one occasion a member of staff was not able to carry out a task that they should have been able to do	Staff were always able to carry out tasks that they should be able to do
My treatment/ procedure/ operation did not always happen on time	My treatment/ procedure/ operation always happened on time

For peer review only - http://bmjopen.bmj.com/site/about/guidelines.xhtml

Stage 2: Revised diagnostic measure reliability

Internal reliability

The revised measure (PMOS-30) demonstrated strong reliability, with a coefficient α of .87. Cronbach's alphas and average inter-item correlations were calculated for domains with three or more items, and inter-item correlations (Pearson) were calculated for domains with two items (Table 4). The Cronbach's alphas for five of the domains ranged from .40 to .75, and for these domains, the average inter-item correlations (Pearson) were all within an acceptable range (.17—.38), as were the inter-item correlations (Pearson) for the two item domains (.23—.42).

For peer review only

For peer review only - http://bmjopen.bmj.com/site/about/guidelines.xhtml

Table 4. PMOS-30 internal reliability domain scores

Domain, n	Items	Cronbach's alpha	Average inter-item correlation*
**Dignity and respect (not linked to any domains), 1**	I was always treated with dignity and respect	N/A	N/A
**Domain 1. Communication and teamworking, 5**	I got answers to all the questions I had about my care	.753	.383
	I always felt staff listened to me about my concerns		
	There was always someone available to deal with every aspect of my care		
	I felt that the attitude of staff towards me was poor (R)		
	Staff worked together as a team here		
**Domain 2. Organisation and care planning, 4**	My medicines were always available	.521	.220
	Staff didn't always know when a doctor changed my plan of care (R)		
	Staff gave me conflicting information about my care (R)		
	When I needed treatment there was always someone available who was trained to do it		
**Domain 3. Access to resources, 3**	Staff/patients waited a long time for porters to arrive (R)	.401	.183
	Staff seemed to struggle to get help when they needed it (R)		
	Equipment and supplies were always available when needed (e.g. hoists, bed pans, walking aids, dressings)		
**Domain 4. Ward type and layout, 7**	Staff were prompt in answering my buzzer	.589	.171
	The ward was able to deal with all my treatment needs		
	Lack of space made it difficult for staff to do their jobs (R)		
	The following aspects of the ward made it uncomfortable for me: Noise levels (R)		
	The following aspects of the ward made it uncomfortable for me: Lighting levels (R)		
	The following aspects of the ward made it uncomfortable for me: Temperature (R)		
	The following aspects of the ward made it uncomfortable for me: Poor cleanliness (R)		
**Domain 5. Information flow, 2**	Information about me that my health care team needed was always available (e.g. drug charts, medical notes, test results)	N/A	.341
	After shift changes staff knew important information about my care		
**Domain 6. Staff roles and responsibilities, 4**	I knew what the different roles of the people caring for me were	.613	.291
	It was clear who was in charge of the ward staff		
	I knew which consultant was in charge of my care		
	I always knew which nurse or nurses were responsible for my care		
**Domain 7. Staff training, 2**	Staff were always able to use the necessary equipment	N/A	.423
	Staff were always able to carry out tasks that they should be able to do		
**Domain 8. Delays, 2**	There were enough staff on the ward to get things done on time	N/A	.238
	My treatment/ procedure/ operation always happened on time		

Notes. (R) indicates item to be reverse coded so higher scores = safer care; N/A = not applicable; * inter-item correlation reported for domains with two items.

For peer review only

For peer review only - http://bmjopen.bmj.com/site/about/guidelines.xhtml

Phase 2

Method

Participants

Stage 1: Shortening exercise brief standalone measure

The shortening exercise to produce the brief standalone measure took a statistical approach, which involved analyses of PMOS data collected using the original measure within the RCT of the PRASE intervention.[11] Details of the sample were presented in study 1, which included a table of sample characteristics (Table 1).

Stage 2: Brief standalone measure reliability and validity

Prior to data collection, members of the YQSR Group's patient panel reviewed the measure resulting from the statistical exercise to ensure it was acceptable (face validity). The subsequent reliability and validity analyses included 165 patient participants who were recruited across nine wards in one hospital trust in the UK between June 2017 and January 2018. The characteristics of the sample are presented in Table 5, and patient eligibility criteria were those reported in the RCT of the PRASE intervention.[11]

Table 5. Characteristics of the patient sample

	**PMOS-10 N = 165**	
**Age mean (SD)**	53.55 (17.11)	
**Gender**	Female = 55.8%<br>Male = 44.2%	
**Length of stay in days mean (SD)**	5.11 (7.99)	
**Ethnicity**	African	1.2%
	Bangladeshi	.6%
	Black African	.6%
	Indian	1.2%
	Irish	.6%
	Other Asian Background	.6%
	Pakistani	7.9%
	White & Black Caribbean	.6%
	White & Asian	1.2%
	White British	83.6%
	Other White Background	1.8%

Note. SD = standard deviation.

Recruitment and informed consent

Patient participants were recruited by a research practitioner and an undergraduate industrial placement student. Posters were placed on the wards and staff were encouraged to inform patients that researchers were recruiting on the ward. Researchers liaised with the nurse in charge to identify patients who had capacity and were considered well enough to

For peer review only - http://bmjopen.bmj.com/site/about/guidelines.xhtml

For peer review only

take part. Researchers then approached patients to give them information about the study, both written (in the form of a participant information sheet) and verbally. If the patient agreed to take part and gave informed consent they were recruited into the study.

Measures

PMOS-10: (10 items).[g]

Friends and Family Test (FFT)[28]: The FFT is a measure of patient experience which asks if people would recommend the services they have used and offers a range of responses (Extremely Likely; Likely; Neither Likely nor Unlikely; Unlikely; Extremely Unlikely - "How likely are you to recommend this ward to friends and family if they needed similar care or treatment?" (asked at the beginning of the PMOS-10 questionnaire). A lower score on this measure indicated more favourable perceptions.

Analysis

Stage 1: Shortening exercise brief standalone measure

Following a method described by Marteau & Bekker[29] PMOS items were ranked in order of magnitude of their item-remainder correlations using Pearson's correlation (corrected item-total)[h]. PMOS-10 items were selected based on the highest corrected item-total correlations across both PMOS-44 and PMOS-30[i]. We also examined how PMOS-10 correlated with PMOS-44 and PMOS-30.

Stage 2: Brief standalone measure reliability and validity

Estimate of sample size

The sample size was informed by both statistical and pragmatic considerations. Ten cases for each observed variable are recommended. A sample size of 165 patients (mean average of 18 per ward) allowed us to meet these requirements.

Validity and reliability analyses

Taking the same approach as the original PMOS validation study, an overall PMOS-10 'positive index' was constructed by summing the number of items that patients responded to by using one of the two positive response options (e.g. strongly agree, or agree) for a

g The 10-item measure (PMOS-10) can be requested via https://www.improvementacademy.org/tools-and-resources/patient-reporting-and-action-for-a-safe-environment.html.
h Missing data were excluded list-wise.
i PMOS-30 refers to items retained in the 30 item measure. This analyses was based on the PRASE RCT data, therefore subsequent rewording and rephrasing of PMOS-30 items was not accounted for.

For peer review only - http://bmjopen.bmj.com/site/about/guidelines.xhtml

positively worded item; strongly disagree, disagree for a negatively worded item). Therefore, patients could have a score of 10 where higher responses equate to more favourable safety perceptions.

Internal reliability

We assessed the internal consistency reliability[j] of PMOS-10 (Cronbach's alpha), and in line with guidance, a reliability coefficient α of >.7 was deemed acceptable.[25, 26]

Convergent validity

To determine the convergent validity of the 10 item questionnaire (i.e. whether the measure converges with an existing related measure), we examined the association between the PMOS-10 'positive index' and the FFT scores using Pearson's correlation[k]. In line with Cohen's guidelines,[30] correlations of .1 were interpreted as a small effect, .3 as medium and .5 as large.

Discriminant validity

A one-way ANOVA[l] was conducted to assess the extent to which PMOS-10 discriminated amongst the nine wards. Ward was entered as a fixed factor, and the PMOS-10 'positive index' as the dependent factor.

Results

Stage 1: Shortening exercise brief standalone measure

Table 6 shows the 10 highest items (based on corrected item-total correlation) that were included in both PMOS-44 and PMOS-30. These items formed PMOS-10. PMOS-10 correlated with the PMOS-44 (r = .90, p = .000) and PMOS-30 (r = .91, p = .000).

j Missing data were excluded list-wise.
k Missing data were excluded list-wise.
l Missing data were excluded list-wise.

For peer review only - http://bmjopen.bmj.com/site/about/guidelines.xhtml

Table 6. Retained items for PMOS-10

Original wording of item in PMOS-44	Item wording in PMOS-30	Domain (original)	PMOS-44 Corrected item-total correlation	PMOS-30 Corrected item-total correlation
I got answers to all the questions I had about my care	I got answers to all the questions I had about my care	Communication and teamworking	.58	.56
A doctor changed my plan of care and other staff didn't know about it	Staff didn't always know when a doctor changed my plan of care	Organisation and care planning	.50	.48
After a shift change staff did not appear to know important information about my care	After shift changes staff knew important information about my care	Information flow	.52	.49
Staff were prompt in answering my buzzer	Staff were prompt in answering my buzzer	Ward type and layout	.49	.51
Sometimes there was no-one available to deal with aspects of my care	There was always someone available to deal with every aspect of my care	Communication and teamworking	.59	.59
On at least one occasion a member of staff was not able to carry out a task that they should have been able to do	Staff were always able to carry out tasks that they should be able to do	Staff training	.54	.52
I felt that the attitude of staff towards me was poor	I felt that the attitude of staff towards me was poor	Communication and teamworking	.50	.50
I have needed treatment and there was no-one available who was trained to do it	When I needed treatment there was always someone available who was trained to do it	Organisation and care planning	.55	.53
I always felt staff listened to me about my concerns	I always felt staff listened to me about my concerns	Communication and teamworking	.56	.53
Staff seemed to struggle to get help when they needed it	Staff seemed to struggle to get help when they needed it	Access to resources	.51	.48

Stage 2: Brief standalone measure reliability and validity

The mean PMOS-10 'positive index' index score for the entire sample was 8.32 (SD = 1.71), meaning that on average patients responded positively to around eight of the PMOS-10 items.

Internal reliability

The PMOS-10 measure demonstrated good reliability, with a coefficient α of .79.

For peer review only - http://bmjopen.bmj.com/site/about/guidelines.xhtml

Convergent validity

A significant negative correlation was established between the PMOS-10 'positive index and the FFT scores (r = -.42, *p* = .000), indicating that the more positive PMOS scores among patients, the more positive FFT scores were.

Discriminant validity

A one-way between-groups ANOVA was performed to assess the extent to which the PMOS positive index discriminated among the nine wards. There was an overall effect of ward (*F* (8, 156) = 2.1, *p* = .035). Tests of between factors using the Tukey HSD test indicated no significant differences between wards.

Discussion

We aimed to produce two revised patient measures of safety that are practical, feasible and simple for patients to complete, responding directly to the need to to enhance patient acceptability of the original measure.[19, 20] In phase 1, a revised shorter measure (PMOS-30) was produced. The shortening exercise (stage 1) resulted in 14 items being removed and the percentage of negatively worded items was reduced from 57% to 33%. Reducing the number of negatively worded items addressed issues around clarity and the potential for unreliable responses, as some previous research suggests that reverse worded items can be contaminated by inattention and confusion.[31] To ensure adequate content validity, we retained a minimum of two items per domain across the nine domains known to contribute to safety incidents in hospitals and which formed the basis of the original measure[13, 14] informed by the YCFF.[15] In addition, and in collaboration with our patient representatives, to establish the face validity of PMOS-30, items were rephrased to facilitate greater patient understanding. The internal reliability of PMOS-30 was established. Five domains demonstrated an acceptable Cronbach's alpha coefficient and average inter-item correlation, or an acceptable inter-item correlation (two item domains): Communication and teamworking; Information flow; Staff roles and responsibilities; Staff training; and Delays. The remaining three domains had acceptable average inter-item correlations, but a poor Cronbach's alpha coefficient: Organisation and care planning; Access to resources; Ward type and layout. Collectively, these findings demonstrate the stability of of the original domains over time, even within a shorter, revised measure and suggests that the conceptual underpinnings and psychometric properties of the original measure have been preserved.

In phase 2, stage 1; we examined the 10 most highly correlated items that were included in both PMOS-44 (original measure) and PMOS-30. These items formed the briefer standalone

For peer review only - http://bmjopen.bmj.com/site/about/guidelines.xhtml

measure (PMOS-10), which was then ratified prior to stage 2 in collaboration with patient representatives to establish face validity. In stage 2, a strong alpha coefficient demonstrated the internal reliability of PMOS-10. To assess convergent validity we examined the association between PMOS-10 and the FFT (which asks if people would recommend the services they have used to others), and a significant negative correlation was established. The correlation was in the expected direction—when PMOS scores were more positive, so were FFT scores. The strength of the correlation (moderate) suggests that although PMOS-10 'converges' with another known measure of care experience from the patient perspective (FFT), it is indeed measuring something different. To determine disciminant validity, we examined whether PMOS-10 was able to discriminate meaningfully at the ward level, with a view to PMOS-10 being used as a tool to identify those wards which might benefit most from further data gathering efforts (i.e PMOS-30) and improvement as part of the PRASE intervention. There was a significant main effect of ward, but no significant differences between wards. Therefore, based on our anlayses of nine wards, we cannot purport that PMOS-10 is able to meaningfully discrimiatate between wards.

There has been substantial research and policy focus on patient involvement in patient safety.[32-34] Patients providing feedback about the safety of their care is one way for patients to be enaged in patient safety.[35] The collection of feedback on patient satisfaction and experiences of care are also commonplace .[4] In terms of safety specifically, there is a wealth of research in support of patients providing feedback about the safety of their care,[8,9] and the PRASE intervention, is the first of its kind to collect patient feedback about safety on an on-going basis to support service improvement. Vincent and colleagues' MMSF[3] emphasised that patients and carers play an essential role in safety monitoring, but are often an underused resource. To move from patients and carers being an underused resource in the monitoring of safety, we need to ensure that the measures we do have to capture their perspective are acceptable to patients and carers, are not burdensome to complete and are reliable and valid. The research presented in this paper responds directly to these needs.

Strengths and limitations

A major strength of phase 1 is the large sample size of 2002 patient participants used in the stage 1 initial shortening exercise analysis which produced PMOS-30. There were also a number of researchers and hospital volunteers with an extensive amount of experience of facilitating PMOS (original measure) data collection and patient representatives involved. This provided a rich source of data regarding potential rephrasing of items; leading to considerable face validity. A further strength is that the reliability testing of PMOS-30 took

For peer review only - http://bmjopen.bmj.com/site/about/guidelines.xhtml

place in a variety of wards across three hospital trusts. Again, for phase 2, the involvement of patient representatives is a key strength of the PMOS-10 development phase.

A limitation of our work is that our studies only included patient participants who could understand English. Therefore, further testing would be required if PMOS-30 and PMOS-10 were to be translated into other languages. In phase 2, although the wards recruited were from a variety of medical and surgical specialities, a limitation of this stage is that only one hospital trust was involved. Finally, the convergent validity of PMOS-10 was only explored with regards to one related indicator (FFT). We chose the FFT due to it being a patient level measure, as opposed to a ward level measure of safety.

Implications for health services researcher, policy and practice

By futher refining an existing measure, the findings from phase 1 respond to previous research which called for the acceptability of PMOS to be enhanced. The length and some content of the original measure were problematic, and posed issues relating to participant fatigue and burden. The output of a highly reliable and valid shortened diagnostic version of PMOS, which retains the psychometric properties and conceptual underpinnings of the original measure, is integral to the wider adoption and ongoing implementation of the PRASE intervention.

The high patient recruitment rate (92%) for PMOS-10 in study phase 2 strengthens the argument that the measure is acceptable to patients, and these findings position PMOS-10 as a reliable and valid brief standalone measure for on-going monitoring of ward safety performance from the patient perspective. To further validate PMOS-10, research is needed to assess the discriminant validity PMOS-10 on larger scale, in addition to exploring associations with other indicators of patient safety.

Conclusion

Two revised, shortened versions of the original PMOS-44 (PMOS-30 and PMOS-10) were produced to capture patient feedback about safety in hospital, and these measures demonstrated acceptable reliability and validity. PMOS-30 was produced to serve a diagnostic function to be used, alongside the PRASE intervention as a whole. PMOS-10 was developed to be used as a brief standalone measure for on-going monitoring of ward safety performance.

For peer review only

For peer review only - http://bmjopen.bmj.com/site/about/guidelines.xhtml

References

1 Pronovost PJ, Goeschel CA, Marsteller JA, Sexton JB, Pham JC, Berenholtz SM. Framework for patient safety research and improvement. Circulation. 2009 Jan 20;119(2):330-7.

2 Pronovost PJ, Wachter RM. Progress in patient safety: a glass fuller than it seems. American Journal of Medical Quality. 2014 Mar;29(2):165-9.

3 Vincent C, Burnett S, Carthey J. The measurement and monitoring of safety: drawing together academic evidence and practical experience to produce a framework for safety measurement and monitoring. The Health Foundation; 2013.

4 Coulter A, Fitzpatrick R, Cornwell J. Measures of patients' experience in hospital: purpose, methods and uses. London: King's Fund; 2009 Jul.

5 Francis R. Report of the Mid Staffordshire NHS Foundation Trust public inquiry: executive summary. The Stationery Office; 2013 Feb 6.

6 Keogh B. Review into the quality of care and treatment provided by 14 hospital trusts in England: overview report. 2013.

7 National Advisory Group on the Safety of Patients in England. A Promise to Learn a Commitment to Act: Improving the Safety of Patients in England. National Advisory Group on the safety of patients in England; 2013.

8 O'Hara JK, Armitage G, Reynolds C, Coulson C, Thorp L, Din I, Watt I, Wright J. How might health services capture patient-reported safety concerns in a hospital setting? An exploratory pilot study of three mechanisms. BMJ Qual Saf. 2016 Feb 4:bmjqs-2015.

9 Ward JK, Armitage G. Can patients report patient safety incidents in a hospital setting? A systematic review. BMJ Qual Saf. 2012 Aug 1;21(8):685-99.Quality & Safety. 2012;21(8):685–99.

10 Armitage G, Moore S, Reynolds C, Laloë PA, Coulson C, McEachan R, Lawton R, Watt I, Wright J, O'Hara J. Patient-reported safety incidents as a new source of patient safety data: an exploratory comparative study in an acute hospital in England. Journal of health services research & policy. 2018 Jan;23(1):36-43.

11 Lawton R, O'Hara J, Sheard L, Armitage G, Cocks K, Buckley H. Corbacho B, Reynolds C, Marsh C, Moore S, Watt I. Can patient involvement improve patient safety? A cluster randomized control trial of the Patient Reporting and Action for a Safe Environment (PRASE) Intervention. BMJ Qual Saf. 2017 Aug 1;26(8):622-31.

12 O'Hara JK, Lawton RJ, Armitage G, et al. The patient reporting and action for a safe environment (PRASE) intervention: a feasibility study. BMC Health Serv Res. 2016;16:676

For peer review only - http://bmjopen.bmj.com/site/about/guidelines.xhtml

13 Giles SJ, Lawton RJ, Din I, McEachan RR. Developing a patient measure of safety (PMOS). BMJ Qual Saf. 2013 Jul 1;22(7):554-62.

14 McEachan RRC, Lawton RJ, O'Hara JK, et al. Developing a reliable and valid patient measure of safety in hospitals (PMOS): a validation study BMJ Qual Saf 2014;23:565-573.

15 Lawton, R., et al., Development of an evidence-based framework of factors contributing to patient safety incidents in hospital settings: a systematic review. BMJ quality & safety, 2012:p. bmjqs-2011-000443.

16 Rolstad S, Adler J, Rydén A. Response burden and questionnaire length: is shorter better? A review and meta-analysis. Value in Health. 2011 Dec 1;14(8):1101-8.

17 Kelly L, Sizmur S, Käsbauer S, King J, Cooper R, Jenkinson C, Graham C. The Relational Aspects of Care Questionnaire: item reduction and scoring using inpatient and accident and emergency data in England. Patient related outcome measures. 2018;9:173.

18 Nielsen MB, Hystad SW, Eid J. The Brief Norwegian Safety Climate Inventory (Brief NORSCI)–Psychometric properties and relationships with shift work, sleep, and health. Safety science. 2016 Mar 1;83:23-30.

19 Louch G, O'hara J, Mohammed MA. A qualitative formative evaluation of a patient-centred patient safety intervention delivered in collaboration with hospital volunteers. Health Expectations. 2017 Oct;20(5):1143-53.

20 Taylor N, Hogden E, Clay-Williams R, Li Z, Lawton R, Braithwaite J. Older, vulnerable patient view: a pilot and feasibility study of the patient measure of safety (PMOS) with patients in Australia. BMJ open. 2016 Jun 1;6(6):e011069.

21 Louch G, Mohammed MA, Hughes L, O'hara J. “Change is what can actually make the tough times better”: A patient-centred patient safety intervention delivered in collaboration with hospital volunteers. Health Expectations. 2019 Feb;22(1):102-13.

22 IBM Corp. IBM SPSS statistics: Version 23. 0. 2016.

23 Guest G, Namey E. Public health research methods. Thousand Oaks, CA: Sage Publications; 2014. p.392.

24 Briggs SR, Cheek JM. The role of factor analysis in the development and evaluation of personality scales. Journal of personality. 1986 Mar;54(1):106-48.

25 Bland JM, Altman DG. Statistics notes: Cronbach's alpha. BMJ. 1997 Feb 22;314(7080):572.

26 Tavakol M, Dennick R. Making sense of Cronbach's alpha. International Journal of Medical Education. 2011;2:53.

For peer review only

For peer review only - http://bmjopen.bmj.com/site/about/guidelines.xhtml

27 Clark LA, Watson D. Constructing validity: Basic issues in objective scale development. Psychological assessment. 1995 Sep;7(3):309.

28 Department of Health. The NHS Friends and Family Test Implementation Guidance. https://www.gov.uk/government/uploads/system/uploads/attachment_data/file/213047/NHS-Friends-and-Family-Test-Implementation-Guidance-v2.pdf (accessed April 2019).

29 Marteau TM, Bekker H. The development of a six-item short-form of the state scale of the Spielberger State—Trait Anxiety Inventory (STAI). British Journal of Clinical Psychology. 1992 Sep 1;31(3):301-6.

30 Cohen J. A power primer. Psychological bulletin. 1992 Jul;112(1):155.

31 Van Sonderen E, Sanderman R, Coyne JC. Ineffectiveness of reverse wording of questionnaire items: Let's learn from cows in the rain. PloS one. 2013 Jul 31;8(7):e68967.

32 Davis RE, Sevdalis N, Vincent CA. Patient involvement in patient safety: How willing are patients to participate?. BMJ Quality & Safety. 2011 Jan 1;20(1):108-14.

33 Vincent CA, Coulter A. Patient safety: what about the patient?. BMJ Quality & Safety. 2002 Mar 1;11(1):76-80.

34 The Health Foundation. Evidence scan: Involving patients in improving safety. London: The Health Foundation. 2013.

35 O'Hara J, Lawton R. At a crossroads? Key challenges and future opportunities for patient involvement in safety. BMJ Quality & Safety. 2016;0:1-4. doi:10.1136/bmjqs-2016-005476

For peer review only

For peer review only - http://bmjopen.bmj.com/site/about/guidelines.xhtml

BMJ Open

Validation of revised Patient Measures of Safety: PMOS-30 and PMOS-10

Journal:	*BMJ Open*
Manuscript ID	bmjopen-2019-031355.R1
Article Type:	Original research
Date Submitted by the Author:	01-Aug-2019
Complete List of Authors:	Louch, Gemma; Bradford Institute for Health Research, Yorkshire Quality and Safety Research Group<br>Reynolds , Caroline ; Bradford Institute for Health Research, Yorkshire Quality and Safety Research Group<br>Moore, Sally; Bradford Institute for Health Research, Yorkshire Quality and Safety Research Group<br>Marsh, Claire; Bradford Institute for Health Research, Yorkshire Quality and Safety Research Group<br>Heyhoe, Jane; Bradford Institute for Health Research, Yorkshire Quality and Safety Research Group<br>Albutt, Abigail; Bradford Institute for Health Research, Yorkshire Quality and Safety Research Group<br>Lawton, Rebecca; University of Leeds, School of Psychology; Bradford Institute for Health Research, Yorkshire Quality and Safety Research Group
**Primary Subject Heading**:	Health services research
Secondary Subject Heading:	Patient-centred medicine
Keywords:	patient safety, patient involvement, psychometrics, patient feedback, acute care

SCHOLARONE™ Manuscripts

Validation of revised Patient Measures of Safety: PMOS-30 and PMOS-10

Gemma Louch[1], Caroline Reynolds[1], Sally Moore[1], Claire Marsh[1], Jane Heyhoe[1], Abigail Albutt[1], Rebecca Lawton[1 2] and on behalf of the Yorkshire Quality and Safety Research Group[1]

[1] *Yorkshire Quality and Safety Research Group, Bradford Institute for Health Research, Bradford Royal Infirmary, Bradford, BD9 6RJ, UK*

[2] *School of Psychology, University of Leeds, Leeds, LS2 9JT, UK*

Correspondence

Gemma Louch: Gemma.Louch@bthft.nhs.uk

Yorkshire Quality and Safety Research Group, Bradford Institute for Health Research, Bradford Royal Infirmary, Bradford, BD9 6RJ, UK

For peer review only

Abstract

Objectives: There is growing evidence that patients can provide feedback on the safety of their care. The 44-item Patient Measure of Safety (PMOS) was developed for this purpose. While valid and reliable, the length of this questionnaire makes it potentially challenging for routine use. Our study aimed to produce revised, shortened versions of PMOS (PMOS-30 and PMOS-10), which retained the psychometric properties of the longer version.

Participants: To produce a shortened diagnostic measure we analysed data from 2002 patients who completed PMOS-44, and examined the reliability of the revised measure (PMOS-30) in a sample of 751 patients. To produce a brief standalone measure, we again analysed data from 2002 patients who completed PMOS-44, and tested the reliability and validity of the brief standalone measure (PMOS-10) in a sample of 165 patients.

Methods: The process of shortening the questionnaire involved a combination of secondary data analysis (e.g. standard deviation and inter-item correlations) and a consensus group exercise to produce PMOS-30 and examine face validity. Analysis of PMOS-30 data examined reliability (e.g. Cronbach's alpha). Further secondary data analysis (i.e.. corrected item-total correlations) produced PMOS-10, and primary data collection assessed its reliability and validity (e.g. Cronbach's alpha, analysis of variance).

Results: Fourteen items were removed to produce PMOS-30 and the percentage of negatively worded items was reduced from 57% to 33%. PMOS-30 demonstrated good internal reliability ($\alpha = .89$). The 10 items with the highest corrected item-total correlations across both PMOS-44 and PMOS-30 composed PMOS-10. PMOS-10 had good internal reliabilty ($\alpha = .79$), demonstrated convergent validity, however, discriminant validity was not established.

Conclusions: Two revised, shortened versions of the original PMOS-44 (PMOS-30 and PMOS-10) were produced to capture patient feedback about safety in hospital. The measures demonstrated good reliability and validity, and preserved the psychometric properties of the original measure.

Keywords: patient feedback, patient safety, patient involvement, acute care, psychometrics

For peer review only - http://bmjopen.bmj.com/site/about/guidelines.xhtml

Strengths and limitations of this study

- Reliability testing of PMOS-30 took place in a variety of wards across three hospital trusts
- The involvement of patient representatives is a key strength of the PMOS-10 development phase
- A limitation of our work is that our studies only included patient participants who could understand English

Background

Measurement has posed an ongoing challenge in patient safety improvement. The difficulties and tensions associated with developing and implementing measures of patient safety are well recognised, and there is a need for measures to be relevant to multiple stakeholders, have a scientific basis, as well as being feasible and usable.[1] Indeed, there have been calls for more time and money to be invested in the science of safety measurement and the implementation of systems to capture such measures in order to understand whether safety is improving.[2] Furthermore, there is recognition that safety cannot be captured in a single measure, and that patients and carers play an essential role in safety monitoring, but are often an underused resource.[3]

The collection of feedback on patient satisfaction and experiences of care is now commonplace,[4] and in the UK, multiple high profile reports have highlighted the importance of listening and responding to patient views about safety.[5-7] There is growing evidence that patients can provide feedback on the safety of their care,[8, 9] and research to suggest that patients can offer a unique perspective on patient safety not captured by other established methods of incident detection.[10] To provide a proactive diagnostic tool which systematically invites the patient perspective on safety, researchers from the UK developed an intervention to provide a theory and evidence-based approach for the collection of hospital in-patient feedback about safety to support service improvement. The Patient Reporting and Action for a Safe Environment (PRASE) intervention[11, 12] includes two measurement tools. The first is a 44-item theory based measure—the Patient Measure of Safety (PMOS),[13, 14] informed by the domains of the Yorkshire Contributory Factors Framework (YCFF).[15] The second is the Patient Incident Reporting Tool (PIRT)[12] which enables patients to report safety concerns and positive experiences.

The original measure included a minimum of two items per domain, across nine domains, known to contribute to safety incidents in hospitals: (1) Communication and teamworking; (2) Organisation and care planning; (3) Access to resources; (4) Ward type and layout; (5)

For peer review only - http://bmjopen.bmj.com/site/about/guidelines.xhtml

Information flow; (6) Staff roles and responsibilities; (7) Staff training; (8) Equipment (design and functioning); (9) Delays. Within a Randomised Controlled Trial (RCT) of the PRASE intervention,[11] PMOS data was collected from over two thousand patient participants. The researchers and research nurses who facilitated this data collection reported that some patients, particularly those who were older or very unwell, struggled with the length of the measure and the phrasing of some of the negatively worded items. Evidence suggests that shorter measures and a reduction in assessment time can lessen the burden on participants and improve response rates and missing data.[16] Therefore, for a large number of questionnaires used in a healthcare context, often short form or brief measures are produced to address this issue with the aim of maintaining acceptable reliability and validity (e.g.[17, 18]).

Whilst in the PRASE RCT[11] researchers were on hand to answer questions and encourage responses, so the above issues did not pose a major problem (response rates were 86%), these issues may result in lower uptake as use becomes more widespread. For example, if data are collected via hospital volunteers (one option that has been tested), difficulties with question wording and/or the length of the measure pose a threat to the reliability and validity of the responses, and may result in missing data. Indeed, in a formative evaluation of the implementation of PRASE with hospital volunteers—volunteers described how the measure was too time consuming for patients to complete, and that its length impacted on the conversation and rapport they were able to build with patients.[19] Furthermore, in recognition of the potential difficulties described above, researchers in Australia have produced a revised version of the measure for use with vulnerable older adult groups.[20]

Objectives

The objectives of this research were to produce two measures that are practical, feasible and simple for hospital inpatients to complete. As it is currently conceived, the PMOS has a diagnostic function. It can be used to identify those factors (see domains above) where attention might most usefully be focused when planning improvement. The first objective therefore was to produce a revised version of PMOS that was shorter with improved acceptability, whilst at the same time preserving the psychometric properties, conceptual underpinnings and diagnostic function of the longer version. This version would continue to be used, alongside the PIRT tool as a basis for the PRASE intervention. Our second objective was to develop a version of PMOS that was much shorter and that could be used as a brief standalone measure for on-going monitoring of ward safety performance not focussed on planning improvement. Potentially this measure could be used to prompt

For peer review only

For peer review only - http://bmjopen.bmj.com/site/about/guidelines.xhtml

additional information gathering effort using the refined diagnostic measure or to monitor safety performance over time.

Patient and public involvement statement

Patients and the public were involved in this study, specifically the Yorkshire Quality and Safety Research (YQSR) Group patient panel and hospital volunteers from Bradford Teaching Hospitals NHS Foundation Trust. More information is provided in the method.

Method

First we focussed on the diagnostic measure and aimed to reduce the number of PMOS items, rephrase items where appropriate, consider face and content validity, and establish the internal reliability of the revised diagnostic measure. Next we focussed on producing a brief standalone measure for on-going monitoring of ward safety performance and establishing its internal reliability, face validity, convergent validity and discriminant validity.

Participants

PMOS-30

The shortening exercise included the analysis of PMOS data collected using the original measure (44 items) within the RCT of the PRASE intervention.[11] Analyses included 2002 patient participants recruited across three hospital trusts and 33 wards in the UK between May 2013 and September 2014. This exercise also included an iterative consensus approach in a series of meetings with researchers from the Yorkshire Quality and Safety Research (YQSR) Group who developed the original measure, and six researchers and research nurses with experience of facilitating the collection of PMOS data in the RCT of the PRASE intervention. These meetings aimed to reduce the number of items in the measure, and considered the phrasing of retained items. At this stage, to establish face validity, we also sought input on item wordings/phrasings from two patient panel representatives (from the YQSR Group's patient panel), and two hospital volunteers involved in a project implementing PRASE in collaboration with hospital volunteers, which we henceforth refer to as the hospital volunteers project[a].[19, 21]

Subsequently the internal reliability of the revised PMOS was tested in a sample of 751 patients. Patients were recruited by hospital volunteers across 22 wards from three hospitals, at three hospital trusts in the UK between September 2015 and May 2016, within

a https://www.health.org.uk/improvement-projects/putting-the-patient-at-the-heart-of-patient-safety.

For peer review only - http://bmjopen.bmj.com/site/about/guidelines.xhtml

the hospital volunteers project. The characteristics of the patient samples are presented in Table 1.

PMOS-10

The shortening exercise to produce a brief standalone measure took a statistical approach, which involved analyses of PMOS data collected using the original measure within the RCT of the PRASE intervention.[11] The characteristics of the patient samples are presented in Table 1

Prior to data collection, members of the YQSR Group's patient panel reviewed the measure resulting from the statistical exercise to ensure it was acceptable (face validity). The subsequent reliability and validity analyses included 165 patient participants who were recruited across nine wards in one hospital trust in the UK between June 2017 and January 2018. Patient eligibility criteria were those reported in the RCT of the PRASE intervention.[11]

Table 1. Characteristics of the patient samples

	**PMOS-44**<br>**N = 2002**<br>**Data collected 2013-2014**		**PMOS-30**<br>**N = 751**<br>**Data collected 2015-2016**		**PMOS-10**<br>**N = 165**<br>**Data collected 2017-2018**	
**Ward type**	Medicine = 41.20%<br>Surgery = 56.60%<br>Mixed = 2.20%		Medicine = 41.10%<br>Surgery = 49.00%<br>Paediatrics = 9.90%		Medicine = 49.70%<br>Surgery = 50.30%	
**Age mean (SD)**	62.48 (17.28)		54.26 (25.07)		53.55 (17.11)	
**Gender**	Female = 50.0%<br>Male = 50.0%		Female = 53.5%<br>Male = 44.7%		Female = 55.2%<br>Male = 44.8%	
**Length of stay in days mean (SD)**	6.94 (12.47)		6.15 (7.57)		5.11 (7.99)	
**Ethnicity**	African	.3%	African	.1%	African	1.2%
	Bangladeshi	.1%	Bangladeshi	.1%	Bangladeshi	.6%
	British	93.5%	British	89.7%	Black African	.6%
	Caribbean	.6%	Indian	1.5%	Indian	1.2%
	Chinese	.2%	Irish	.7%	Irish	.6%
	Indian	.5%	Other	1.9%	Other Asian Background	.6%
	Irish	.6%	Other Background	.1%	Pakistani	7.9%
	Other	1.2%	Other Ethnic Background	.5%	White & Black Caribbean	.6%
	Other Background	.3%	Pakistani	4.3%	White & Asian	1.2%
	Other Mixed Background	.2%	White & Asian	.1%	White British	83.6%
	Pakistani	1.0%	Missing	.9%	Other White Background	1.8%
	White & Black Caribbean	.2%				
	Missing	1.1%				

Note. SD = standard deviation.

For peer review only - http://bmjopen.bmj.com/site/about/guidelines.xhtml

Recruitment and informed consent

PMOS-30

Patients were invited to complete the questionnaire by hospital volunteers within a quality improvement project; therefore informed consent was not required. The hospital volunteer training package was based on the training for researchers and research nurses who facilitated patient data collection within the RCT of the PRASE intervention.[11] More information regarding the procedure for hospital volunteers visiting wards has been published previously[19].

PMOS-10

Patients were recruited by a research nurse and an undergraduate industrial placement student. Posters were placed on wards and staff were encouraged to inform patients that researchers were recruiting. Researchers liaised with the nurse in charge to identify patients who had capacity and were considered well enough to take part. Researchers then approached patients to give them information about the study, both written (in the form of a participant information sheet) and verbally. If the patient agreed to take part and gave informed consent they were recruited into the study.

Measures[b]

The response options for PMOS-44 (44 items), PMOS-30 (30 items) and PMOS-10 (10 items) are as follows: 1 = Strongly Disagree, 2 = Disagree, 3 = Neither agree or disagree, 4 = Agree, 5 = Strongly Agree, not applicable, and I prefer not to answer. Responses are recoded such that a high score indicates more favourable perceptions. PMOS-44 and PMOS-30 questionnaires were completed using a laptop/handheld device. PMOS-10 questionnaires were collected on paper. For all versions of the PMOS questionnaire, patients could complete the questionnaire themselves, or completion could be facilitated (Researchers/research nurses – PMOS-44; Hospital volunteers – PMOS-30; research nurse/industrial placement student – PMOS-10).

To examine the convergent validity of PMOS-10, the Friends and Family Test (FFT)[22] question was asked at the beginning of the PMOS-10 questionnaire. The FFT is a one item measure of patient experience which asks if people would recommend the services they have used and offers a range of responses (Extremely Likely; Likely; Neither Likely nor Unlikely; Unlikely; Extremely Unlikely - “How likely are you to

b PMOS-44, PMOS-30, PMOS-10 and ‘easy read’ versions which have subsequently been produced can be requested via https://www.improvementacademy.org/tools-and-resources/patient-reporting-and-action-for-a-safe-environment.html (freely available).

For peer review only - http://bmjopen.bmj.com/site/about/guidelines.xhtml

recommend this ward to friends and family if they needed similar care or treatment?" A lower score on this measure indicated more favourable perceptions.

Analysis

All analyses were performed using SPSS Version 23.[23] Our analytical approach was informed by the original PMOS validation study,[14] For PMOS data, if a patient responded 'not applicable' or 'I prefer not to answer' this was treated as missing data as these responses do not contribute to PMOS scoring. These two additional response options accounted for 87.39% of missing data points for PMOS-44, 72.39% for PMOS-30, and 99.44% for PMOS-10. The PMOS-30 and PMOS-10 shortening analyses only included PMOS-44 data where collection was facilitated by a researcher/research nurse, so we could be more confident in the reliability of responses. The PMOS-30 and PMOS-10 reliability and validity analyses included both facilitated and self-completed responses. All missing data were excluded list-wise[c], and the retained number of cases differed dependent on the type of analysis performed. There were the following numbers of complete PMOS responses across the datasets: PMOS-44 (n = 1119); PMOS-30 (n = 347); PMOS-10 (n = 119), there was no missing data for the FFT question.

Estimate of sample size

For the PMOS-30 and PMOS-10 reliability and validity analyses the sample sizes were informed by both statistical and pragmatic considerations, based on a minimum subject to item ratio of at least 10:1.[24 25]

PMOS-30

Researchers and research nurses who collected PMOS data from patients within the RCT of the PRASE intervention met to discuss the content of the original measure. Potential items for removal were highlighted, for example, based on patient difficulties in understanding of the item or patient feedback that items were repetitive. Prior to a series of consensus meetings analyses were agreed, the results of which would be brought to the consensus meetings. These analyses included item response variation (standard deviation) and inter-item correlations using Pearson's correlation.

Research suggests that for a 5-point scale items which demonstrate reasonable variability (standard deviation of 1.00 or higher) are potential items to retain and that items with limited variance are candidates for removal.[26] On this basis, items demonstrating low variability (i.e.

c The characteristics of patients with missing data were consistent with the larger samples.

For peer review only - http://bmjopen.bmj.com/site/about/guidelines.xhtml

<.7) were identified as candidate items for removal, and in terms of the level of overlap between items (possible multicollinearity), high inter-item correlations highlighted candidate items for removal, i.e. higher than .5.[27]

Subsequently, in a series of consensus meetings with the wider research group, items which had been identified as problematic, based on either researcher/research nurse perceptions or the item analyses, were discussed in greater detail. For the purpose of construct validity, the group considered each potential item for removal, and focussed on whether each item reliably tapped what it was intended to measure. For the purpose of content validity we also endeavoured to ensure a minimum of two items per domain were retained. We used multiple criteria, rather than only a statistical approach when considering items for removal to safeguard against key items being removed.

Reliability analyses

We examined the internal consistency reliability (Cronbach's alpha) of PMOS-30 data collected from 751 hospital in-patients within the hospital volunteers project. In line with guidance, a reliability coefficient α of >.7 was deemed acceptable.[28, 29] For domains with three or more items we examined the internal consistency reliability in terms of original measure's domain structure i.e. the domains that form the basis of the feedback to support service improvement in the PRASE intervention, and assessed the average inter-item correlations using the recommended values range of .15 to .50.[30] For two item domains we assessed inter-item correlations.

PMOS-10

Following a method described by Marteau & Bekker[31] PMOS items were ranked in order of magnitude of their item-remainder correlations using Pearson's correlation (corrected item-total). PMOS-10 items were selected based on the highest corrected item-total correlations across both PMOS-44 and PMOS-30[d]. We also examined how PMOS-10 correlated with PMOS-44 and PMOS-30.

Validity and reliability analyses

Taking the same approach as the original PMOS validation study, an overall PMOS-10 'positive index' was constructed by summing the number of items that patients responded to by using one of the two positive response options (Strongly Agree or Agree) for a positively worded item and (Strongly Disagree or disagree) for a negatively worded item. Therefore,

d PMOS-30 refers to items retained in the 30 item measure. This analyses was based on the PRASE RCT data, therefore subsequent rewording and rephrasing of PMOS-30 items were not accounted for.

For peer review only - http://bmjopen.bmj.com/site/about/guidelines.xhtml

patients could have a score of 10 where higher responses equate to more favourable safety perceptions. If patients had missing responses, the ‘positive index’ was constructed from the available data.

Internal reliability

We assessed the internal consistency reliability of PMOS-10 (Cronbach’s alpha), and in line with guidance, a reliability coefficient α of >.7 was deemed acceptable.[28, 29]

Convergent validity

To determine the convergent validity of the 10 item questionnaire (i.e. whether the measure converges with an existing related measure), we examined the association between the PMOS-10 ‘positive index’ and the FFT scores using Pearson's correlation. In line with Cohen's guidelines,[32] correlations of .1 were interpreted as a small effect, .3 as medium and .5 as large.

Discriminant validity

A one-way ANOVA was conducted to assess the extent to which PMOS-10 discriminated amongst the nine wards. Ward was entered as a fixed factor, and the PMOS-10 ‘positive index’ as the dependent factor.

Results

PMOS-30

Of the 44 items in the original measure, 14 items were removed (32%). Table 2 presents the rationale for the removal of each item. Eight items were removed due to both statistical reasons and researcher/research nurse feedback, five items were removed due to researcher/research nurse feedback alone, and one item was removed due to statistical reasons alone. To facilitate the likelihood of reliable and valid responses, we also reduced the quantity of negatively phrased items in the revised measure. In the original measure the proportion of negatively phrased items was 57% (25 items); in the revised measure this was reduced to 33% (10 items). See Table 3 or the rephrasing of retained items.

Internal reliability

The revised measure (PMOS-30) demonstrated strong reliability, with a coefficient α of .89. Cronbach’s alphas and average inter-item correlations were calculated for domains with three or more items, and inter-item correlations (Pearson) were calculated for domains with two items (Table 4). The Cronbach’s alphas for five of the domains ranged from .40 to .75, and for these domains, the average inter-item correlations (Pearson) were all within an

For peer review only - http://bmjopen.bmj.com/site/about/guidelines.xhtml

acceptable range (.17—.38), as were the inter-item correlations (Pearson) for the two item domains (.23—.42).

For peer review only

For peer review only - http://bmjopen.bmj.com/site/about/guidelines.xhtml

Table 2. Rationale for item removal

**Item removed**	**Rationale**	
	Consensus group discussion	***Item analysis***
The following aspects of the ward made it difficult for staff to do their jobs: Lighting levels	Researchers/research nurses highlighted that frequently patients did not respond to this item with regards to themselves, and therefore the item was often not answered as intended i.e. how lighting levels affect staff	Low variability SD = .59
Staff were always able to get advice from other teams about my care if needed	Researchers/research nurses highlighted that frequently patients did not respond to this item in terms of what the item is attempting to tap i.e. specialist staff etc.	Low variability SD = .67 High inter-item correlations with 'I got answers to all the questions I had about my care' (.51)
When staff talked about my care with others the information they shared was correct	Researchers/research nurses highlighted that frequently patients did not respond to this item in terms of what the item is attempting to tap i.e. information sharing between staff	Low variability SD = .61
The following aspects of the ward made it difficult for staff to do their jobs: Position of nurses' station	Researchers/research nurses highlighted that participants often struggled to respond to this item from the patient perspective	Low variability SD = .71
There was equipment that staff found difficult to use (e.g. monitoring equipment, beds, hoists)	Researchers/research nurses highlighted that frequently patients reported that it would be more appropriate for staff to respond to this item	Low variability SD = .64
Staff were kept waiting for my test results	Researchers/research nurses felt this issue was covered elsewhere in the measure i.e. 'Information about me that my health care team needed was always available'	
The following aspects of the ward made it uncomfortable for me: Lack of Space	Researchers/research nurses felt that lack of space was assessed elsewhere in the measure, albeit in relation to staff rather than patients. 'The following aspects of the ward made it difficult for staff to do their jobs: Lack of space'	High inter-item correlations with 'The following aspects of the ward made it difficult for staff to do their jobs: Lack of space' (.51)

For peer review only

Item removed	Rationale	
	Consensus group discussion	***Item analysis***
Staff always seemed to know what they were meant to be doing	Researchers/research nurses highlighted that patients often felt they could not answer this. Researchers/researcher nurses also felt there was conceptual overlap with another item i.e. 'I got answers to all the questions I had about my care'	Low variability SD = .59
Staff always agreed about my treatment/care	Researchers/research nurses felt there was conceptual overlap with another item i.e. 'Staff gave me different information about my care'	Low variability SD = .60
Nurses were always able to get help from other staff when they asked for it	Researchers/research nurses felt there was conceptual overlap with another item i.e. 'Staff seemed to struggle to get help when they needed it'	Low variability SD = .59
I knew who to go to if I needed to ask a question	Researchers/research nurses felt other items capture this issue	
The following aspects of the ward made it difficult for staff to do their jobs: Clutter & untidiness		Low variability SD = .67
Equipment needed for my care was always working properly	Researchers/research nurses highlighted that frequently patients reported that it would be more appropriate for staff to respond to this item	Low variability SD = .71
The following aspects of the ward made it uncomfortable for me: Other - Please specify	Researchers/research nurses felt this item could be removed due to low response rate	

Note. SD = standard deviation.

For peer review only

For peer review only - http://bmjopen.bmj.com/site/about/guidelines.xhtml

Table 3. Retained items rephrasing

PMOS-44 item	PMOS-30 item
Sometimes there was no-one available to deal with aspects of my care	There was always someone available to deal with every aspect of my care
Staff did not work together as a team here	Staff worked together as a team here
The drugs I have been prescribed were always available in the hospital	My medicines were always available
A doctor changed my plan of care and other staff didn't know about it	Staff didn't always know when a doctor changed my plan of care
Staff gave me different information about my care	Staff gave me conflicting information about my care
I have needed treatment and there was no-one available who was trained to do it	When I needed treatment there was always someone available who was trained to do it
Equipment and supplies were not always available when needed (e.g. hoists, bed pans, drugs)	Equipment and supplies were always available when needed (e.g. hoists, bed pans, walking aids, dressings)
I was on a ward that was not able to deal with my treatment needs	The ward was able to deal with all my treatment needs
After a shift change staff did not appear to know important information about my care	After shift changes staff knew important information about my care
It was clear who was in charge of the staff	It was clear who was in charge of the ward staff
I always knew which nurse was responsible for my care	I always knew which nurse or nurses were responsible for my care
On at least one occasion a member of staff was not able to use the necessary equipment	Staff were always able to use the necessary equipment
On at least one occasion a member of staff was not able to carry out a task that they should have been able to do	Staff were always able to carry out tasks that they should be able to do
My treatment/ procedure/ operation did not always happen on time	My treatment/ procedure/ operation always happened on time

For peer review only - http://bmjopen.bmj.com/site/about/guidelines.xhtml

Table 4. PMOS-30 internal reliability domain scores

Domain, number of items	Items	Cronbach's alpha	Average inter-item correlation*
**Dignity and respect (not linked to any domains), 1**	I was always treated with dignity and respect	N/A	N/A
**Domain 1. Communication and teamworking, 5**	I got answers to all the questions I had about my care	.753	.383
	I always felt staff listened to me about my concerns		
	There was always someone available to deal with every aspect of my care		
	I felt that the attitude of staff towards me was poor (R)		
	Staff worked together as a team here		
**Domain 2. Organisation and care planning, 4**	My medicines were always available	.521	.220
	Staff didn't always know when a doctor changed my plan of care (R)		
	Staff gave me conflicting information about my care (R)		
	When I needed treatment there was always someone available who was trained to do it		
**Domain 3. Access to resources, 3**	Staff/patients waited a long time for porters to arrive (R)	.401	.183
	Staff seemed to struggle to get help when they needed it (R)		
	Equipment and supplies were always available when needed (e.g. hoists, bed pans, walking aids, dressings)		
**Domain 4. Ward type and layout, 7**	Staff were prompt in answering my buzzer	.589	.171
	The ward was able to deal with all my treatment needs		
	Lack of space made it difficult for staff to do their jobs (R)		
	The following aspects of the ward made it uncomfortable for me: Noise levels (R)		
	The following aspects of the ward made it uncomfortable for me: Lighting levels (R)		
	The following aspects of the ward made it uncomfortable for me: Temperature (R)		
	The following aspects of the ward made it uncomfortable for me: Poor cleanliness (R)		
**Domain 5. Information flow, 2**	Information about me that my health care team needed was always available (e.g. drug charts, medical notes, test results)	N/A	.341*
	After shift changes staff knew important information about my care		
**Domain 6. Staff roles and responsibilities, 4**	I knew what the different roles of the people caring for me were	.613	.292
	It was clear who was in charge of the ward staff		
	I knew which consultant was in charge of my care		
	I always knew which nurse or nurses were responsible for my care		
**Domain 7. Staff training, 2**	Staff were always able to use the necessary equipment	N/A	.423*
	Staff were always able to carry out tasks that they should be able to do		
**Domain 8. Delays, 2**	There were enough staff on the ward to get things done on time	N/A	.238*
	My treatment/ procedure/ operation always happened on time		

Notes. (R) indicates item to be reverse coded so higher scores = safer care; N/A = not applicable; * inter-item correlation reported for domains with two items.

For peer review only - http://bmjopen.bmj.com/site/about/guidelines.xhtml

PMOS-10

Table 5 shows the 10 highest items (based on corrected item-total correlation) that were included in both PMOS-44 and PMOS-30. These items formed PMOS-10. PMOS-10 correlated with the PMOS-44 (r = .90, *p* = .000) and PMOS-30 (r = .91, *p* = .000).

Table 5. Retained items for PMOS-10

**Original wording of item in PMOS-44**	**Item wording in PMOS-30**	**Domain (PMOS-44 and PMOS-30)**	**PMOS-44 Corrected item-total correlation**	**PMOS-30 Corrected item-total correlation**
I got answers to all the questions I had about my care	I got answers to all the questions I had about my care	Communication and teamworking	.58	.56
A doctor changed my plan of care and other staff didn't know about it	Staff didn't always know when a doctor changed my plan of care	Organisation and care planning	.50	.48
After a shift change staff did not appear to know important information about my care	After shift changes staff knew important information about my care	Information flow	.52	.49
Staff were prompt in answering my buzzer	Staff were prompt in answering my buzzer	Ward type and layout	.49	.51
Sometimes there was no-one available to deal with aspects of my care	There was always someone available to deal with every aspect of my care	Communication and teamworking	.59	.59
On at least one occasion a member of staff was not able to carry out a task that they should have been able to do	Staff were always able to carry out tasks that they should be able to do	Staff training	.54	.52
I felt that the attitude of staff towards me was poor	I felt that the attitude of staff towards me was poor	Communication and teamworking	.50	.50
I have needed treatment and there was no-one available who was trained to do it	When I needed treatment there was always someone available who was trained to do it	Organisation and care planning	.55	.53
I always felt staff listened to me about my concerns	I always felt staff listened to me about my concerns	Communication and teamworking	.56	.53
Staff seemed to struggle to get help when they needed it	Staff seemed to struggle to get help when they needed it	Access to resources	.51	.48

The mean PMOS-10 'positive index' index score for the entire sample was 8.37 (SD = 1.65), meaning that on average patients responded positively to around eight of the PMOS-10 items. The recruitment response rate was 92%.

For peer review only - http://bmjopen.bmj.com/site/about/guidelines.xhtml

Internal reliability

The PMOS-10 measure demonstrated good reliability, with a coefficient α of .79.

Convergent validity

A significant negative correlation was established between the PMOS-10 'positive index and the FFT scores (r = -.36, *p* = .000), indicating that the more positive PMOS scores among patients, the more positive FFT scores were.

Discriminant validity

A one-way between-groups ANOVA was performed to assess the extent to which the PMOS positive index discriminated among the nine wards. There was no significant main effect of ward (F (8, 156) = 1.59, p = .130).

Discussion

We aimed to produce two revised patient measures of safety that are practical, feasible and simple for patients to complete, responding directly to the need to enhance patient acceptability of the original measure.[19, 20] PMOS-30 included less negatively worded items compared to PMOS-44 (37% as opposed to 57%), and the internal reliability of PMOS-30 was established. Reducing the number of negatively worded items addressed the potential for unreliable responses, as previous research suggests that reverse worded items can be contaminated by inattention and confusion.[33] We retained a minimum of two items per domain across the nine domains known to contribute to safety incidents in hospitals and which formed the basis of the original measure[13, 14] informed by the YCFF.[15] In addition, and in collaboration with our patient representatives, items were rephrased to facilitate greater patient understanding.. One domain demonstrated an acceptable Cronbach's alpha coefficient and acceptable average inter-item correlation: Communication and teamworking. Four domains did not demonstrate an acceptable Cronbach's alpha coefficient but demonstrated an acceptable average inter-item correlation: Organisation and care planning, Access to resources, Ward type and layout, and Staff roles and responsibilities. Three domains (two item domains) demonstrated acceptable inter-item correlations: Information flow, Staff training, and Delays. The reduction in the number of items may have resulted in lowering the reliability statistics of the domains, however this is to be expected as a reliability coefficient generally increases as the number of items increases.[29] Interestingly the domains which performed poorest (e.g. Ward type and layout; Access to resources) also performed poorest in the original PMOS validation study[14]. Collectively, these findings

For peer review only - http://bmjopen.bmj.com/site/about/guidelines.xhtml

demonstrate the stability of the original domains over time, even within a shorter, revised measure and suggests that the conceptual underpinnings and psychometric properties of the original measure have been preserved.

The 10 most highly correlated items that were included in both PMOS-44 (original measure) and PMOS-30 formed the briefer standalone measure (PMOS-10), which was ratified in collaboration with patient representatives to establish face validity. A good alpha coefficient demonstrated the internal reliability of PMOS-10, and convergent validity was established. The strength of the correlation (moderate) suggests that although PMOS-10 'converges' with another known measure of care experience from the patient perspective (FFT), it is indeed measuring something different. To determine disciminant validity, we examined whether PMOS-10 was able to discriminate meaningfully at the ward level, with a view to PMOS-10 being used as a tool to identify those wards which might benefit most from further data gathering efforts (i.e PMOS-30) and improvement as part of the PRASE intervention. There was no significant main effect of ward and no significant differences between wards. Therefore, based on our anlayses of nine wards, we cannot purport that PMOS-10 is able to meaningfully discrimiatate between wards.

There has been substantial research and policy focus on patient involvement in patient safety.[34-37] Patients providing feedback about the safety of their care is one way for patients to be enaged in patient safety.[38] The collection of feedback on patient satisfaction and experiences of care are also commonplace.[4] In terms of safety specifically, there is a wealth of research in support of patients providing feedback about the safety of their care,[8,9] and the PRASE intervention, is the first of its kind to collect patient feedback about safety on an on-going basis to support service improvement. Vincent and colleagues'[3] emphasised that patients and carers play an essential role in safety monitoring, but are often an underused resource. To move from patients and carers being an underused resource in the monitoring of safety, we need to ensure that the measures we do have to capture their perspective are acceptable to patients and carers, are not burdensome to complete and are reliable and valid. The research presented in this paper responds directly to these needs.

Strengths and limitations

A strength of the development of PMOS-30 is the involvement of six researchers/research nurses with an extensive amount of experience of facilitating PMOS (original measure) data collection, and the involvement of two hospital volunteers and two patient representatives. This provided a rich source of data regarding potential rephrasing of items; leading to considerable face validity. A further strength is that the reliability testing of PMOS-30 took

For peer review only - http://bmjopen.bmj.com/site/about/guidelines.xhtml

place in a variety of wards across three hospital trusts. The involvement of two patient represenatives in the development of PMOS-10 is a key strength.

A limitation of our work is that our studies only included patient participants who could understand English. Therefore, further testing would be required if PMOS-30 and PMOS-10 were to be translated into other languages. For PMOS-30, some of the domains failing to achieve the Cronbach's alpha recommendation of .70 is a limitation, even though the average inter-item and inter-item correlations were within the optimum range. For PMOS-10, although the wards recruited were from a variety of medical and surgical specialities, a limitation is the involvement of only one hospital trust. A further limitation is that disciminant validity was not established for PMOS-10.

Implications for health services researcher, policy and practice

By futher refining an existing measure, the findings respond to previous research which called for the acceptability of PMOS to be enhanced. The length and some content of the original measure were problematic, and posed issues relating to participant fatigue and burden. The output of a highly reliable and valid shortened diagnostic version of PMOS, which retains the psychometric properties and conceptual underpinnings of the original measure, is integral to the wider adoption and ongoing implementation of the PRASE intervention. The high recruitment response rate (92%) for PMOS-10 strengthens the argument that the measure is acceptable to patients, and these findings position PMOS-10 as a reliable and valid brief standalone measure for on-going monitoring of ward safety performance from the patient perspective. To further validate PMOS-10, research is needed to assess the discriminant validity PMOS-10 on larger scale, in addition to further exploring convergent validity by examining associations with other indicators of patient safety.

Conclusion

Two revised, shortened versions of the original PMOS-44 (PMOS-30 and PMOS-10) were produced to capture patient feedback about safety in hospital, and these measures demonstrated acceptable reliability and validity. PMOS-30 was produced to serve a diagnostic function to be used, alongside the PRASE intervention as a whole. PMOS-10 was developed to be used as a brief standalone measure for on-going monitoring of ward safety performance.

For peer review only - http://bmjopen.bmj.com/site/about/guidelines.xhtml

Twiter: @Gemma_Louch

Competing interests: None declared.
Acknowledgements: We would like to thank the Yorkshire Quality and Safety Research (YQSR) Group patient panel for reviewing the PMOS-10 measure, and we would like to thank the two members of the panel and two hospital volunteers for their time and input into revising the PMOS-30 measure. We would also like to thank the patient participants and the staff on the wards for their contribution to the research.
Funding statement: The research was supported by The Health Foundation, the National Institute for Health Research (NIHR) Yorkshire and Humber Patient Safety Translational Research Centre (NIHR YH PSTRC), and NIHR CLAHRC Yorkshire and Humber www.clahrc-yh.nihr.ac.uk. The views expressed in this article are those of the author(s) and not necessarily those of The Health Foundation, the NHS, the NIHR, or the Department of Health and Social Care.
Author contribution: GL led on the development of PMOS-30, carried out the related analyses and drafted the manuscript. JH carried out the PMOS-10 shortening statistical analyses. CR led on the PMOS-10 data collection, and GL, AA and CR carried out the related analyses. SM and CM supported the studies at all stages, in particular the patient and public involvement and engagement element of this work. RL provided guidance and input at all stages. All authors provided comments and approved the final version.
Ethics approval: Ethical approvals were obtained from the University of Bradford, Humanities, Social and Health Sciences Research Ethics Panel, and from the East Midlands - Leicester Central Research Ethics Committee.
Data sharing statement: The data that support the findings of this study are available from the corresponding author, GL, upon reasonable request.

For peer review only

References

1 Pronovost PJ, Goeschel CA, Marsteller JA, Sexton JB, Pham JC, Berenholtz SM. Framework for patient safety research and improvement. Circulation. 2009 Jan 20;119(2):330-7.

2 Pronovost PJ, Wachter RM. Progress in patient safety: a glass fuller than it seems. American Journal of Medical Quality. 2014 Mar;29(2):165-9.

3 Vincent C, Burnett S, Carthey J. The measurement and monitoring of safety: drawing together academic evidence and practical experience to produce a framework for safety measurement and monitoring. The Health Foundation; 2013.

4 Coulter A, Fitzpatrick R, Cornwell J. Measures of patients' experience in hospital: purpose, methods and uses. London: King's Fund; 2009 Jul.

5 Francis R. Report of the Mid Staffordshire NHS Foundation Trust public inquiry: executive summary. The Stationery Office; 2013 Feb 6.

6 Keogh B. Review into the quality of care and treatment provided by 14 hospital trusts in England: overview report. 2013.

7 National Advisory Group on the Safety of Patients in England. A Promise to Learn a Commitment to Act: Improving the Safety of Patients in England. National Advisory Group on the safety of patients in England; 2013.

8 O'Hara JK, Armitage G, Reynolds C, Coulson C, Thorp L, Din I, Watt I, Wright J. How might health services capture patient-reported safety concerns in a hospital setting? An exploratory pilot study of three mechanisms. BMJ Qual Saf. 2016 Feb 4:bmjqs-2015.

9 Ward JK, Armitage G. Can patients report patient safety incidents in a hospital setting? A systematic review. BMJ Qual Saf. 2012 Aug 1;21(8):685-99.Quality & Safety. 2012;21(8):685–99.

10 Armitage G, Moore S, Reynolds C, Laloë PA, Coulson C, McEachan R, Lawton R, Watt I, Wright J, O'Hara J. Patient-reported safety incidents as a new source of patient safety data: an exploratory comparative study in an acute hospital in England. Journal of health services research & policy. 2018 Jan;23(1):36-43.

11 Lawton R, O'Hara J, Sheard L, Armitage G, Cocks K, Buckley H. Corbacho B, Reynolds C, Marsh C, Moore S, Watt I. Can patient involvement improve patient safety? A cluster randomized control trial of the Patient Reporting and Action for a Safe Environment (PRASE) Intervention. BMJ Qual Saf. 2017 Aug 1;26(8):622-31.

12 O'Hara JK, Lawton RJ, Armitage G, et al. The patient reporting and action for a safe environment (PRASE) intervention: a feasibility study. BMC Health Serv Res. 2016;16:676

For peer review only - http://bmjopen.bmj.com/site/about/guidelines.xhtml

13 Giles SJ, Lawton RJ, Din I, McEachan RR. Developing a patient measure of safety (PMOS). BMJ Qual Saf. 2013 Jul 1;22(7):554-62.

14 McEachan RRC, Lawton RJ, O'Hara JK, et al. Developing a reliable and valid patient measure of safety in hospitals (PMOS): a validation study BMJ Qual Saf 2014;23:565-573.

15 Lawton, R., et al., Development of an evidence-based framework of factors contributing to patient safety incidents in hospital settings: a systematic review. BMJ quality & safety, 2012:p. bmjqs-2011-000443.

16 Rolstad S, Adler J, Rydén A. Response burden and questionnaire length: is shorter better? A review and meta-analysis. Value in Health. 2011 Dec 1;14(8):1101-8.

17 Kelly L, Sizmur S, Käsbauer S, King J, Cooper R, Jenkinson C, Graham C. The Relational Aspects of Care Questionnaire: item reduction and scoring using inpatient and accident and emergency data in England. Patient related outcome measures. 2018;9:173.

18 Nielsen MB, Hystad SW, Eid J. The Brief Norwegian Safety Climate Inventory (Brief NORSCI)–Psychometric properties and relationships with shift work, sleep, and health. Safety science. 2016 Mar 1;83:23-30.

19 Louch G, O'hara J, Mohammed MA. A qualitative formative evaluation of a patient-centred patient safety intervention delivered in collaboration with hospital volunteers. Health Expectations. 2017 Oct;20(5):1143-53.

20 Taylor N, Hogden E, Clay-Williams R, Li Z, Lawton R, Braithwaite J. Older, vulnerable patient view: a pilot and feasibility study of the patient measure of safety (PMOS) with patients in Australia. BMJ open. 2016 Jun 1;6(6):e011069.

21 Louch G, Mohammed MA, Hughes L, O'hara J. "Change is what can actually make the tough times better": A patient-centred patient safety intervention delivered in collaboration with hospital volunteers. Health Expectations. 2019 Feb;22(1):102-13.

22 Department of Health. The NHS Friends and Family Test Implementation Guidance. https://www.gov.uk/government/uploads/system/uploads/attachment_data/file/213047/NHS-Friends-and-Family-Test-Implementation-Guidance-v2.pdf (accessed April 2019).

23 IBM Corp. IBM SPSS statistics: Version 23. 0. 2016.

24 Hatcher, L. (1994). A Step-by-Step Approach to Using the SAS® System for Factor Analysis and Structural Equation Modeling. Cary, N.C.: SAS Instittute, Inc.

25 Kyriazos TA. Applied psychometrics: Sample size and sample power considerations in factor analysis (EFA, CFA) and SEM in general. Psychology. 2018 Aug 2;9(08):2207.

26 Guest G, Namey E. Public health research methods. Thousand Oaks, CA: Sage Publications; 2014. p.392.

For peer review only - http://bmjopen.bmj.com/site/about/guidelines.xhtml

27 Briggs SR, Cheek JM. The role of factor analysis in the development and evaluation of personality scales. Journal of personality. 1986 Mar;54(1):106-48.

28 Bland JM, Altman DG. Statistics notes: Cronbach's alpha. BMJ. 1997 Feb 22;314(7080):572.

29 Tavakol M, Dennick R. Making sense of Cronbach's alpha. International Journal of Medical Education. 2011;2:53.

30 Clark LA, Watson D. Constructing validity: Basic issues in objective scale development. Psychological assessment. 1995 Sep;7(3):309.

31 Marteau TM, Bekker H. The development of a six-item short-form of the state scale of the Spielberger State—Trait Anxiety Inventory (STAI). British Journal of Clinical Psychology. 1992 Sep 1;31(3):301-6.

32 Cohen J. A power primer. Psychological bulletin. 1992 Jul;112(1):155.

33 Van Sonderen E, Sanderman R, Coyne JC. Ineffectiveness of reverse wording of questionnaire items: Let's learn from cows in the rain. PloS one. 2013 Jul 31;8(7):e68967.

34 Davis RE, Sevdalis N, Vincent CA. Patient involvement in patient safety: How willing are patients to participate?. BMJ Quality & Safety. 2011 Jan 1;20(1):108-14.

35 Vincent CA, Coulter A. Patient safety: what about the patient?. BMJ Quality & Safety. 2002 Mar 1;11(1):76-80.

36 O'Hara J, Lawton R. At a crossroads? Key challenges and future opportunities for patient involvement in safety. BMJ Quality & Safety. 2016;0:1-4. doi:10.1136/bmjqs-2016-005476

37 World Health Organization. Patients for Patient Safety Partnerships for Safer Health Care. Geneva: World Health Organization WHO. 2013. Available from: http://www.who.int/patientsafety/patients_for_patient/PFPS_brochure_2013.pdf

38 The Health Foundation. Evidence scan: Involving patients in improving safety. London: The Health Foundation. 2013.

For peer review only - http://bmjopen.bmj.com/site/about/guidelines.xhtml

COSMIN Study Design checklist for Patient-reported outcome measurement instruments*

General recommendations for the design of a study on measurement properties	Y/N
Research aim	
1 Provide a clear research aim, including (1) the name and version of the PROM, (2) the target population, and (3) the measurement properties of interest	Y
PROM	
2 Provide a clear description of the construct to be measured	Y
3 Provide a clear description of the development process of the PROM, including a description of the target population for which the PROM was developed	Y and in original validation study.
4 The origin of the construct should be clear: provide a theory, conceptual framework (i.e. reflective or formative model) or disease model used or clear rationale to define the construct to be measured	Y and in original validation study.
5 Provide a clear description of the structure of the PROM (i.e. the number of items and subscales included in the PROM, instructions given and response options) and its scoring algorithm	Y
6 Provide a clear description of existing evidence on the quality of the PROM	Y
7 Provide a clear description of the context of use*	Y
Target population	
8 Provide a clear description of in- and exclusion criteria to select patients, e.g. in terms of disease condition and characteristics like age, gender, language or country, and setting (e.g. general population, primary care or hospital/rehabilitation care)	Y
9 Provide a clear description of the method used to select the patients for the study (e.g. convenience, consecutive, or random)	Y
10 Describe whether the selected sample is representing the target population in which the PROM will be used in terms of age, gender, important disease characteristics (e.g. severity, status, duration)	Y
**Content validity**	
Design requirements	
1 From the perspective of the patients: use an appropriate method for assessing (1) the relevance of each item for the patients' experience with the condition, AND (2) the comprehensiveness of the PROM, AND (3) the comprehensibility of the PROM instructions, items, response options, and recall period	Y
2 From the perspective of professionals: use	Covered in development of

For peer review only

an appropriate method for assessing (1) the relevance of each item for the construct of interest, AND (2) the comprehensiveness of the PROM	original measure.
3 Include professionals from all relevant disciplines	N/A
4 Evaluate each item in an appropriate number of patients or professionals For qualitative studies For quantitative (survey) studies	Y
5 Use skilled group moderators or interviewers	Y
6 Base the group meetings or interviews on an appropriate topic or interview guide	Y
7 Record and transcribe verbatim the group meetings or interviews	N
Analyses	
8 Use an appropriate approach to analyse the data	Y
9 Involve at least two researchers in the analysis	Y
**Internal consistency**	
**Design requirements**	
1 Check whether a scale or a subscale is unidimensional	Covered in original validation study.
2 Perform the analysis in a sample with an appropriate number of patients (taking into account expected number of missing values)	Y
3 Provide a clear description of how missing items will be handled	Y
Statistical methods	
4 For continuous scores: calculate Cronbach's alpha or Omega for each unidimensional scale or subscale	Y
5 For dichotomous scores: calculate Cronbach's alpha or KR-20 for each unidimensional scale or subscale	N/A
6 For IRT-based scores: calculate standard error of theta (SE (θ)) or reliability coefficient of estimated latent trait value (index of (subject or item) separation) for each unidimensional scale or subscale	N/A
**Hypotheses testing for construct validity**	
**A. Comparison with other outcome measurement instruments (convergent validity)**	
Design requirements	
1 Formulate hypotheses about expected relationships between the PROM under study and other outcome measurement instrument(s)	Y
2 Provide a clear description of the construct(s) measured by the comparator instrument(s)	Y
3 Use comparator instrument(s) with sufficient measurement properties	Y
4 Perform the analysis in a sample with an appropriate number of patients (taking into account expected number of missing values)	Y
5 Use an appropriate time schedule for assessments of the PROM of interest and comparison instruments	Y
Statistical methods	

For peer review only

6 Use statistical methods that are appropriate for the hypotheses to be tested	Y
7 Provide a clear description of how missing items will be handled	Y
**B. Comparison between subgroups (discriminative or known-groups validity)**	
Design requirements	
1 Formulate hypotheses regarding mean differences between subgroups	Y
2 Provide an adequate description of important characteristics of the subgroups, such as disease or demographic characteristics	Y
3 Perform the analysis in a sample with an appropriate number of patients (taking into account expected number of missing values)	Y
Statistical methods	
4 Use statistical methods that are appropriate for the hypotheses to be tested	Y
5 Provide a clear description of how missing items will be handled	Y

*Only selected components are included relevant to the study.

For peer review only

BMJ Open

Validation of revised Patient Measures of Safety: PMOS-30 and PMOS-10

Journal:	*BMJ Open*
Manuscript ID	bmjopen-2019-031355.R2
Article Type:	Original research
Date Submitted by the Author:	13-Sep-2019

Complete List of Authors:	Louch, Gemma; Bradford Institute for Health Research, Yorkshire Quality and Safety Research Group<br>Reynolds , Caroline ; Bradford Institute for Health Research, Yorkshire Quality and Safety Research Group<br>Moore, Sally; Bradford Institute for Health Research, Yorkshire Quality and Safety Research Group<br>Marsh, Claire; Bradford Institute for Health Research, Yorkshire Quality and Safety Research Group<br>Heyhoe, Jane; Bradford Institute for Health Research, Yorkshire Quality and Safety Research Group<br>Albutt, Abigail; Bradford Institute for Health Research, Yorkshire Quality and Safety Research Group<br>Lawton, Rebecca; University of Leeds, School of Psychology; Bradford Institute for Health Research, Yorkshire Quality and Safety Research Group

**Primary Subject Heading**:	Health services research
Secondary Subject Heading:	Patient-centred medicine
Keywords:	patient safety, patient involvement, psychometrics, patient feedback, acute care

SCHOLARONE™
Manuscripts

Validation of revised Patient Measures of Safety: PMOS-30 and PMOS-10

Gemma Louch[1], Caroline Reynolds[1], Sally Moore[1], Claire Marsh[1], Jane Heyhoe[1], Abigail Albutt[1], Rebecca Lawton[1 2] and on behalf of the Yorkshire Quality and Safety Research Group[1]

[1] *Yorkshire Quality and Safety Research Group, Bradford Institute for Health Research, Bradford Royal Infirmary, Bradford, BD9 6RJ, UK*

[2] *School of Psychology, University of Leeds, Leeds, LS2 9JT, UK*

Correspondence

Gemma Louch: Gemma.Louch@bthft.nhs.uk

Yorkshire Quality and Safety Research Group, Bradford Institute for Health Research, Bradford Royal Infirmary, Bradford, BD9 6RJ, UK

For peer review only - http://bmjopen.bmj.com/site/about/guidelines.xhtml

Abstract

Objectives: There is growing evidence that patients can provide feedback on the safety of their care. The 44-item Patient Measure of Safety (PMOS) was developed for this purpose. While valid and reliable, the length of this questionnaire makes it potentially challenging for routine use. Our study aimed to produce revised, shortened versions of PMOS (PMOS-30 and PMOS-10), which retained the psychometric properties of the longer version.

Participants: To produce a shortened diagnostic measure we analysed data from 2002 patients who completed PMOS-44, and examined the reliability of the revised measure (PMOS-30) in a sample of 751 patients. To produce a brief standalone measure, we again analysed data from 2002 patients who completed PMOS-44, and tested the reliability and validity of the brief standalone measure (PMOS-10) in a sample of 165 patients.

Methods: The process of shortening the questionnaire involved a combination of secondary data analysis (e.g. standard deviation and inter-item correlations) and a consensus group exercise to produce PMOS-30 and examine face validity. Analysis of PMOS-30 data examined reliability (e.g. Cronbach's alpha). Further secondary data analysis (i.e.. corrected item-total correlations) produced PMOS-10, and primary data collection assessed its reliability and validity (e.g. Cronbach's alpha, analysis of variance).

Results: Fourteen items were removed to produce PMOS-30 and the percentage of negatively worded items was reduced from 57% to 33%. PMOS-30 demonstrated good internal reliability ($\alpha = .89$). The 10 items with the highest corrected item-total correlations across both PMOS-44 and PMOS-30 composed PMOS-10. PMOS-10 had good internal reliabilty ($\alpha = .79$), demonstrated convergent validity, however, discriminant validity was not established.

Conclusions: Two revised, shortened versions of the original PMOS-44 (PMOS-30 and PMOS-10) were produced to capture patient feedback about safety in hospital. The measures demonstrated good reliability and validity, and preserved the psychometric properties of the original measure.

Keywords: patient feedback, patient safety, patient involvement, acute care, psychometrics

For peer review only - http://bmjopen.bmj.com/site/about/guidelines.xhtml

Strengths and limitations of this study

- Reliability testing of PMOS-30 took place in a variety of wards across three hospital trusts
- The involvement of patient representatives is a key strength of the PMOS-10 development phase
- A limitation of our work is that our studies only included patient participants who could understand English

Background

Measurement has posed an ongoing challenge in patient safety improvement. The difficulties and tensions associated with developing and implementing measures of patient safety are well recognised, and there is a need for measures to be relevant to multiple stakeholders, have a scientific basis, as well as being feasible and usable.[1] Indeed, there have been calls for more time and money to be invested in the science of safety measurement and the implementation of systems to capture such measures in order to understand whether safety is improving.[2] Furthermore, there is recognition that safety cannot be captured in a single measure, and that patients and carers play an essential role in safety monitoring, but are often an underused resource.[3]

The collection of feedback on patient satisfaction and experiences of care is now commonplace,[4] and in the UK, multiple high profile reports have highlighted the importance of listening and responding to patient views about safety.[5-7] There is growing evidence that patients can provide feedback on the safety of their care,[8, 9] and research to suggest that patients can offer a unique perspective on patient safety not captured by other established methods of incident detection.[10] To provide a proactive diagnostic tool which systematically invites the patient perspective on safety, researchers from the UK developed an intervention to provide a theory and evidence-based approach for the collection of hospital in-patient feedback about safety to support service improvement. The Patient Reporting and Action for a Safe Environment (PRASE) intervention[11, 12] includes two measurement tools. The first is a 44-item theory based measure—the Patient Measure of Safety (PMOS),[13, 14] informed by the domains of the Yorkshire Contributory Factors Framework (YCFF).[15] The second is the Patient Incident Reporting Tool (PIRT)[12] which enables patients to report safety concerns and positive experiences.

The original measure included a minimum of two items per domain, across nine domains, known to contribute to safety incidents in hospitals: (1) Communication and teamworking; (2) Organisation and care planning; (3) Access to resources; (4) Ward type and layout; (5)

For peer review only - http://bmjopen.bmj.com/site/about/guidelines.xhtml

Information flow; (6) Staff roles and responsibilities; (7) Staff training; (8) Equipment (design and functioning); (9) Delays. Within a Randomised Controlled Trial (RCT) of the PRASE intervention,[11] PMOS data was collected from over two thousand patient participants. The researchers and research nurses who facilitated this data collection reported that some patients, particularly those who were older or very unwell, struggled with the length of the measure and the phrasing of some of the negatively worded items. Evidence suggests that shorter measures and a reduction in assessment time can lessen the burden on participants and improve response rates and missing data.[16] Therefore, for a large number of questionnaires used in a healthcare context, often short form or brief measures are produced to address this issue with the aim of maintaining acceptable reliability and validity (e.g.[17, 18]).

Whilst in the PRASE RCT[11] researchers were on hand to answer questions and encourage responses, so the above issues did not pose a major problem (response rates were 86%), these issues may result in lower uptake as use becomes more widespread. For example, if data are collected via hospital volunteers (one option that has been tested), difficulties with question wording and/or the length of the measure pose a threat to the reliability and validity of the responses, and may result in missing data. Indeed, in a formative evaluation of the implementation of PRASE with hospital volunteers—volunteers described how the measure was too time consuming for patients to complete, and that its length impacted on the conversation and rapport they were able to build with patients.[19] Furthermore, in recognition of the potential difficulties described above, researchers in Australia have produced a revised version of the measure for use with vulnerable older adult groups.[20]

Objectives

The objectives of this research were to produce two measures that are practical, feasible and simple for hospital inpatients to complete. As it is currently conceived, the PMOS has a diagnostic function. It can be used to identify those factors (see domains above) where attention might most usefully be focused when planning improvement. The first objective therefore was to produce a revised version of PMOS that was shorter with improved acceptability, whilst at the same time preserving the psychometric properties, conceptual underpinnings and diagnostic function of the longer version. This version would continue to be used, alongside the PIRT tool as a basis for the PRASE intervention. Our second objective was to develop a version of PMOS that was much shorter and that could be used as a brief standalone measure for on-going monitoring of ward safety performance not focussed on planning improvement. Potentially this measure could be used to prompt

For peer review only - http://bmjopen.bmj.com/site/about/guidelines.xhtml

additional information gathering effort using the refined diagnostic measure or to monitor safety performance over time.

Patient and public involvement statement

Patients and the public were involved in this study in the shortening and rewording of the questionnaires. More information is provided in the method.

Method

First we focussed on the diagnostic measure and aimed to reduce the number of PMOS items, rephrase items where appropriate, consider face and content validity, and establish the internal reliability of the revised diagnostic measure. Next we focussed on producing a brief standalone measure for on-going monitoring of ward safety performance and establishing its internal reliability, face validity, convergent validity and discriminant validity.

Participants

PMOS-30

The shortening exercise included the analysis of PMOS data collected using the original measure (44 items) within the RCT of the PRASE intervention.[11] Analyses included 2002 patient participants recruited across three hospital trusts and 33 wards in the UK between May 2013 and September 2014. This exercise also included an iterative consensus approach in a series of meetings with researchers from the Yorkshire Quality and Safety Research (YQSR) Group who developed the original measure, and six researchers and research nurses with experience of facilitating the collection of PMOS data in the RCT of the PRASE intervention. These meetings aimed to reduce the number of items in the measure, and considered the phrasing of retained items. At this stage, to establish face validity, we also sought input on item wordings/phrasings from two patient panel representatives (from the YQSR Group's patient panel), and two hospital volunteers involved in a project implementing PRASE in collaboration with hospital volunteers, which we henceforth refer to as the hospital volunteers project[19, 21]

Subsequently the internal reliability of the revised PMOS was tested in a sample of 751 patients. Patients were recruited by hospital volunteers across 22 wards from three hospitals, at three hospital trusts in the UK between September 2015 and May 2016, within the hospital volunteers project. The characteristics of the patient samples are presented in Table 1.

For peer review only - http://bmjopen.bmj.com/site/about/guidelines.xhtml

PMOS-10

The shortening exercise to produce a brief standalone measure took a statistical approach, which involved analyses of PMOS data collected using the original measure within the RCT of the PRASE intervention.[11] The characteristics of the patient samples are presented in Table 1

Prior to data collection, members of the YQSR Group's patient panel reviewed the measure resulting from the statistical exercise to ensure it was acceptable (face validity). The subsequent reliability and validity analyses included 165 patient participants who were recruited across nine wards in one hospital trust in the UK between June 2017 and January 2018. Patient eligibility criteria were those reported in the RCT of the PRASE intervention.[11]

Table 1. Characteristics of the patient samples

	**PMOS-44**<br>**N = 2002**<br>**Data collected 2013-2014**		**PMOS-30**<br>**N = 751**<br>**Data collected 2015-2016**		**PMOS-10**<br>**N = 165**<br>**Data collected 2017-2018**	
**Ward type**	Medicine = 41.20%<br>Surgery = 56.60%<br>Mixed = 2.20%		Medicine = 41.10%<br>Surgery = 49.00%<br>Paediatrics = 9.90%		Medicine = 49.70%<br>Surgery = 50.30%	
**Age median, range**	65, 87		60, 100		57, 71	
**Gender**	Female = 50.0%<br>Male = 50.0%		Female = 53.5%<br>Male = 44.7%		Female = 55.2%<br>Male = 44.8%	
**Length of stay in days median, range**	3, 167		4, 90		3, 49	
**Ethnicity**	African	.3%	African	.1%	African	1.2%
	Bangladeshi	.1%	Bangladeshi	.1%	Bangladeshi	.6%
	British	93.5%	British	89.7%	Black African	.6%
	Caribbean	.6%	Indian	1.5%	Indian	1.2%
	Chinese	.2%	Irish	.7%	Irish	.6%
	Indian	.5%	Other	1.9%	Other Asian Background	.6%
	Irish	.6%	Other Background	.1%	Pakistani	7.9%
	Other	1.2%	Other Ethnic Background	.5%	White & Black Caribbean	.6%
	Other Background	.3%	Pakistani	4.3%	White & Asian	1.2%
	Other Mixed Background	.2%	White & Asian	.1%	White British	83.6%
	Pakistani	1.0%	Missing	.9%	Other White Background	1.8%
	White & Black Caribbean	.2%				
	Missing	1.1%				

For peer review only

For peer review only - http://bmjopen.bmj.com/site/about/guidelines.xhtml

Recruitment and informed consent

PMOS-30

Patients were invited to complete the questionnaire by hospital volunteers within a quality improvement project; therefore informed consent was not required. The hospital volunteer training package was based on the training for researchers and research nurses who facilitated patient data collection within the RCT of the PRASE intervention.[11] More information regarding the procedure for hospital volunteers visiting wards has been published previously[19].

PMOS-10

Patients were recruited by a research nurse and an undergraduate industrial placement student. Posters were placed on wards and staff were encouraged to inform patients that researchers were recruiting. Researchers liaised with the nurse in charge to identify patients who had capacity and were considered well enough to take part. Researchers then approached patients to give them information about the study, both written (in the form of a participant information sheet) and verbally. If the patient agreed to take part and gave informed consent they were recruited into the study.

Measures[a]

The response options for PMOS-44 (44 items), PMOS-30 (30 items) and PMOS-10 (10 items) are as follows: 1 = Strongly Disagree, 2 = Disagree, 3 = Neither agree or disagree, 4 = Agree, 5 = Strongly Agree, not applicable, and I prefer not to answer. Responses are recoded such that a high score indicates more favourable perceptions. PMOS-44 and PMOS-30 questionnaires were completed using a laptop/handheld device. PMOS-10 questionnaires were collected on paper. For all versions of the PMOS questionnaire, patients could complete the questionnaire themselves, or completion could be facilitated (Researchers/research nurses – PMOS-44; Hospital volunteers – PMOS-30; research nurse/industrial placement student – PMOS-10).

To examine the convergent validity of PMOS-10, the Friends and Family Test (FFT)[22] question was asked at the beginning of the PMOS-10 questionnaire. The FFT is a one item measure of patient experience which asks if people would recommend the services they have used and offers a range of responses (Extremely Likely; Likely; Neither Likely nor Unlikely; Unlikely; Extremely Unlikely - “How likely are you to

a PMOS-44, PMOS-30, PMOS-10 and ‘easy read’ versions which have subsequently been produced can be requested via https://www.improvementacademy.org/tools-and-resources/patient-reporting-and-action-for-a-safe-environment.html (freely available).

For peer review only - http://bmjopen.bmj.com/site/about/guidelines.xhtml

recommend this ward to friends and family if they needed similar care or treatment?" A lower score on this measure indicated more favourable perceptions.

Analysis

All analyses were performed using SPSS Version 23.[23] Our analytical approach was informed by the original PMOS validation study,[14] For PMOS data, if a patient responded 'not applicable' or 'I prefer not to answer' this was treated as missing data as these responses do not contribute to PMOS scoring. These two additional response options accounted for 87.39% of missing data points for PMOS-44, 72.39% for PMOS-30, and 99.44% for PMOS-10. The PMOS-30 and PMOS-10 shortening analyses only included PMOS-44 data where collection was facilitated by a researcher/research nurse, so we could be more confident in the reliability of responses. The PMOS-30 and PMOS-10 reliability and validity analyses included both facilitated and self-completed responses. All missing data were excluded list-wise[b], and the retained number of cases differed dependent on the type of analysis performed. There were the following numbers of complete PMOS responses across the datasets: PMOS-44 (n = 1119); PMOS-30 (n = 347); PMOS-10 (n = 119), there was no missing data for the FFT question.

Estimate of sample size

For the PMOS-30 and PMOS-10 reliability and validity analyses the sample sizes were informed by both statistical and pragmatic considerations, based on a minimum subject to item ratio of at least 10:1.[24 25]

PMOS-30

Researchers and research nurses who collected PMOS data from patients within the RCT of the PRASE intervention met to discuss the content of the original measure. Potential items for removal were highlighted, for example, based on patient difficulties in understanding of the item or patient feedback that items were repetitive. Prior to a series of consensus meetings analyses were agreed, the results of which would be brought to the consensus meetings. These analyses included item response variation (standard deviation) and inter-item correlations using Pearson's correlation.

Research suggests that for a 5-point scale items which demonstrate reasonable variability (standard deviation of 1.00 or higher) are potential items to retain and that items with limited variance are candidates for removal.[26] On this basis, items demonstrating low variability (i.e.

b The characteristics of patients with missing data were consistent with the larger samples.

For peer review only - http://bmjopen.bmj.com/site/about/guidelines.xhtml

<.7) were identified as candidate items for removal, and in terms of the level of overlap between items (possible multicollinearity), high inter-item correlations highlighted candidate items for removal, i.e. higher than .5.[27]

Subsequently, in a series of consensus meetings with the wider research group, items which had been identified as problematic, based on either researcher/research nurse perceptions or the item analyses, were discussed in greater detail. For the purpose of construct validity, the group considered each potential item for removal, and focussed on whether each item reliably tapped what it was intended to measure. For the purpose of content validity we also endeavoured to ensure a minimum of two items per domain were retained. We used multiple criteria, rather than only a statistical approach when considering items for removal to safeguard against key items being removed.

Reliability analyses

We examined the internal consistency reliability (Cronbach's alpha) of PMOS-30 data collected from 751 hospital in-patients within the hospital volunteers project. In line with guidance, a reliability coefficient α of >.7 was deemed acceptable.[28, 29] For domains with three or more items we examined the internal consistency reliability in terms of original measure's domain structure i.e. the domains that form the basis of the feedback to support service improvement in the PRASE intervention, and assessed the average inter-item correlations using the recommended values range of .15 to .50.[30] For two item domains we assessed inter-item correlations.

PMOS-10

Following a method described by Marteau & Bekker[31] PMOS items were ranked in order of magnitude of their item-remainder correlations using Pearson's correlation (corrected item-total). PMOS-10 items were selected based on the highest corrected item-total correlations across both PMOS-44 and PMOS-30[c]. We also examined how PMOS-10 correlated with PMOS-44 and PMOS-30.

Validity and reliability analyses

Taking the same approach as the original PMOS validation study, an overall PMOS-10 'positive index' was constructed by summing the number of items that patients responded to by using one of the two positive response options (Strongly Agree or Agree) for a positively worded item and (Strongly Disagree or disagree) for a negatively worded item. Therefore,

c PMOS-30 refers to items retained in the 30 item measure. This analyses was based on the PRASE RCT data, therefore subsequent rewording and rephrasing of PMOS-30 items were not accounted for.

For peer review only - http://bmjopen.bmj.com/site/about/guidelines.xhtml

patients could have a score of 10 where higher responses equate to more favourable safety perceptions. If patients had missing responses, the 'positive index' was constructed from the available data.

Internal reliability

We assessed the internal consistency reliability of PMOS-10 (Cronbach's alpha), and in line with guidance, a reliability coefficient α of >.7 was deemed acceptable.[28, 29]

Convergent validity

To determine the convergent validity of the 10 item questionnaire (i.e. whether the measure converges with an existing related measure), we examined the association between the PMOS-10 'positive index' and the FFT scores using Pearson's correlation. In line with Cohen's guidelines,[32] correlations of .1 were interpreted as a small effect, .3 as medium and .5 as large.

Discriminant validity

A one-way ANOVA was conducted to assess the extent to which PMOS-10 discriminated amongst the nine wards. Ward was entered as a fixed factor, and the PMOS-10 'positive index' as the dependent factor.

Results

PMOS-30

Of the 44 items in the original measure, 14 items were removed (32%). Table 2 presents the rationale for the removal of each item. Eight items were removed due to both statistical reasons and researcher/research nurse feedback, five items were removed due to researcher/research nurse feedback alone, and one item was removed due to statistical reasons alone. To facilitate the likelihood of reliable and valid responses, we also reduced the quantity of negatively phrased items in the revised measure. In the original measure the proportion of negatively phrased items was 57% (25 items); in the revised measure this was reduced to 33% (10 items). See Table 3 or the rephrasing of retained items.

Internal reliability

The revised measure (PMOS-30) demonstrated strong reliability, with a coefficient α of .89. Cronbach's alphas and average inter-item correlations were calculated for domains with three or more items, and inter-item correlations (Pearson) were calculated for domains with two items (Table 4). The Cronbach's alphas for five of the domains ranged from .40 to .75, and for these domains, the average inter-item correlations (Pearson) were all within an

For peer review only - http://bmjopen.bmj.com/site/about/guidelines.xhtml

acceptable range (.17—.38), as were the inter-item correlations (Pearson) for the two item domains (.23—.42).

For peer review only

For peer review only - http://bmjopen.bmj.com/site/about/guidelines.xhtml

Table 2. Rationale for item removal

**Item removed**	**Rationale**	
	Consensus group discussion	***Item analysis***
The following aspects of the ward made it difficult for staff to do their jobs: Lighting levels	Researchers/research nurses highlighted that frequently patients did not respond to this item with regards to themselves, and therefore the item was often not answered as intended i.e. how lighting levels affect staff	Low variability SD = .59
Staff were always able to get advice from other teams about my care if needed	Researchers/research nurses highlighted that frequently patients did not respond to this item in terms of what the item is attempting to tap i.e. specialist staff etc.	Low variability SD = .67<br>High inter-item correlations with 'I got answers to all the questions I had about my care' (.51)
When staff talked about my care with others the information they shared was correct	Researchers/research nurses highlighted that frequently patients did not respond to this item in terms of what the item is attempting to tap i.e. information sharing between staff	Low variability SD = .61
The following aspects of the ward made it difficult for staff to do their jobs: Position of nurses' station	Researchers/research nurses highlighted that participants often struggled to respond to this item from the patient perspective	Low variability SD = .71
There was equipment that staff found difficult to use (e.g. monitoring equipment, beds, hoists)	Researchers/research nurses highlighted that frequently patients reported that it would be more appropriate for staff to respond to this item	Low variability SD = .64
Staff were kept waiting for my test results	Researchers/research nurses felt this issue was covered elsewhere in the measure i.e. 'Information about me that my health care team needed was always available'	
The following aspects of the ward made it uncomfortable for me: Lack of Space	Researchers/research nurses felt that lack of space was assessed elsewhere in the measure, albeit in relation to staff rather than patients.<br>'The following aspects of the ward made it difficult for staff to do their jobs: Lack of space'	High inter-item correlations with 'The following aspects of the ward made it difficult for staff to do their jobs: Lack of space' (.51)

For peer review only - http://bmjopen.bmj.com/site/about/guidelines.xhtml

**Item removed**	**Rationale**	
	Consensus group discussion	***Item analysis***
Staff always seemed to know what they were meant to be doing	Researchers/research nurses highlighted that patients often felt they could not answer this. Researchers/researcher nurses also felt there was conceptual overlap with another item i.e. 'I got answers to all the questions I had about my care'	Low variability SD = .59
Staff always agreed about my treatment/care	Researchers/research nurses felt there was conceptual overlap with another item i.e. 'Staff gave me different information about my care'	Low variability SD = .60
Nurses were always able to get help from other staff when they asked for it	Researchers/research nurses felt there was conceptual overlap with another item i.e. 'Staff seemed to struggle to get help when they needed it'	Low variability SD = .59
I knew who to go to if I needed to ask a question	Researchers/research nurses felt other items capture this issue	
The following aspects of the ward made it difficult for staff to do their jobs: Clutter & untidiness		Low variability SD = .67
Equipment needed for my care was always working properly	Researchers/research nurses highlighted that frequently patients reported that it would be more appropriate for staff to respond to this item	Low variability SD = .71
The following aspects of the ward made it uncomfortable for me: Other - Please specify	Researchers/research nurses felt this item could be removed due to low response rate	

Note. SD = standard deviation.

For peer review only - http://bmjopen.bmj.com/site/about/guidelines.xhtml

Table 3. Retained items rephrasing

PMOS-44 item	PMOS-30 item
Sometimes there was no-one available to deal with aspects of my care	There was always someone available to deal with every aspect of my care
Staff did not work together as a team here	Staff worked together as a team here
The drugs I have been prescribed were always available in the hospital	My medicines were always available
A doctor changed my plan of care and other staff didn't know about it	Staff didn't always know when a doctor changed my plan of care
Staff gave me different information about my care	Staff gave me conflicting information about my care
I have needed treatment and there was no-one available who was trained to do it	When I needed treatment there was always someone available who was trained to do it
Equipment and supplies were not always available when needed (e.g. hoists, bed pans, drugs)	Equipment and supplies were always available when needed (e.g. hoists, bed pans, walking aids, dressings)
I was on a ward that was not able to deal with my treatment needs	The ward was able to deal with all my treatment needs
After a shift change staff did not appear to know important information about my care	After shift changes staff knew important information about my care
It was clear who was in charge of the staff	It was clear who was in charge of the ward staff
I always knew which nurse was responsible for my care	I always knew which nurse or nurses were responsible for my care
On at least one occasion a member of staff was not able to use the necessary equipment	Staff were always able to use the necessary equipment
On at least one occasion a member of staff was not able to carry out a task that they should have been able to do	Staff were always able to carry out tasks that they should be able to do
My treatment/ procedure/ operation did not always happen on time	My treatment/ procedure/ operation always happened on time

For peer review only - http://bmjopen.bmj.com/site/about/guidelines.xhtml

Table 4. PMOS-30 internal reliability domain scores

Domain, number of items	Items	Cronbach's alpha	Average inter-item correlation*
**Dignity and respect (not linked to any domains), 1**	I was always treated with dignity and respect	N/A	N/A
**Domain 1. Communication and teamworking, 5**	I got answers to all the questions I had about my care	.753	.383
	I always felt staff listened to me about my concerns		
	There was always someone available to deal with every aspect of my care		
	I felt that the attitude of staff towards me was poor (R)		
	Staff worked together as a team here		
**Domain 2. Organisation and care planning, 4**	My medicines were always available	.521	.220
	Staff didn't always know when a doctor changed my plan of care (R)		
	Staff gave me conflicting information about my care (R)		
	When I needed treatment there was always someone available who was trained to do it		
**Domain 3. Access to resources, 3**	Staff/patients waited a long time for porters to arrive (R)	.401	.183
	Staff seemed to struggle to get help when they needed it (R)		
	Equipment and supplies were always available when needed (e.g. hoists, bed pans, walking aids, dressings)		
**Domain 4. Ward type and layout, 7**	Staff were prompt in answering my buzzer	.589	.171
	The ward was able to deal with all my treatment needs		
	Lack of space made it difficult for staff to do their jobs (R)		
	The following aspects of the ward made it uncomfortable for me: Noise levels (R)		
	The following aspects of the ward made it uncomfortable for me: Lighting levels (R)		
	The following aspects of the ward made it uncomfortable for me: Temperature (R)		
	The following aspects of the ward made it uncomfortable for me: Poor cleanliness (R)		
**Domain 5. Information flow, 2**	Information about me that my health care team needed was always available (e.g. drug charts, medical notes, test results)	N/A	.341*
	After shift changes staff knew important information about my care		
**Domain 6. Staff roles and responsibilities, 4**	I knew what the different roles of the people caring for me were	.613	.292
	It was clear who was in charge of the ward staff		
	I knew which consultant was in charge of my care		
	I always knew which nurse or nurses were responsible for my care		
**Domain 7. Staff training, 2**	Staff were always able to use the necessary equipment	N/A	.423*
	Staff were always able to carry out tasks that they should be able to do		
**Domain 8. Delays, 2**	There were enough staff on the ward to get things done on time	N/A	.238*
	My treatment/ procedure/ operation always happened on time		

Notes. (R) indicates item to be reverse coded so higher scores = safer care; N/A = not applicable; * inter-item correlation reported for domains with two items.

For peer review only - http://bmjopen.bmj.com/site/about/guidelines.xhtml

PMOS-10

Table 5 shows the 10 highest items (based on corrected item-total correlation) that were included in both PMOS-44 and PMOS-30. These items formed PMOS-10. PMOS-10 correlated with the PMOS-44 (r = .90, *p* = .000) and PMOS-30 (r = .91, *p* = .000).

Table 5. Retained items for PMOS-10

**Original wording of item in PMOS-44**	**Item wording in PMOS-30**	**Domain (PMOS-44 and PMOS-30)**	**PMOS-44 Corrected item-total correlation**	**PMOS-30 Corrected item-total correlation**
I got answers to all the questions I had about my care	I got answers to all the questions I had about my care	Communication and teamworking	.58	.56
A doctor changed my plan of care and other staff didn't know about it	Staff didn't always know when a doctor changed my plan of care	Organisation and care planning	.50	.48
After a shift change staff did not appear to know important information about my care	After shift changes staff knew important information about my care	Information flow	.52	.49
Staff were prompt in answering my buzzer	Staff were prompt in answering my buzzer	Ward type and layout	.49	.51
Sometimes there was no-one available to deal with aspects of my care	There was always someone available to deal with every aspect of my care	Communication and teamworking	.59	.59
On at least one occasion a member of staff was not able to carry out a task that they should have been able to do	Staff were always able to carry out tasks that they should be able to do	Staff training	.54	.52
I felt that the attitude of staff towards me was poor	I felt that the attitude of staff towards me was poor	Communication and teamworking	.50	.50
I have needed treatment and there was no-one available who was trained to do it	When I needed treatment there was always someone available who was trained to do it	Organisation and care planning	.55	.53
I always felt staff listened to me about my concerns	I always felt staff listened to me about my concerns	Communication and teamworking	.56	.53
Staff seemed to struggle to get help when they needed it	Staff seemed to struggle to get help when they needed it	Access to resources	.51	.48

The mean PMOS-10 'positive index' index score for the entire sample was 8.37 (SD = 1.65), meaning that on average patients responded positively to around eight of the PMOS-10 items. The recruitment response rate was 92%.

For peer review only - http://bmjopen.bmj.com/site/about/guidelines.xhtml

Internal reliability

The PMOS-10 measure demonstrated good reliability, with a coefficient α of .79.

Convergent validity

A significant negative correlation was established between the PMOS-10 ‘positive index and the FFT scores (r = -.36, *p* = .000), indicating that the more positive PMOS scores among patients, the more positive FFT scores were.

Discriminant validity

A one-way between-groups ANOVA was performed to assess the extent to which the PMOS positive index discriminated among the nine wards. There was no significant main effect of ward (F (8, 156) = 1.59, p = .130).

Discussion

We aimed to produce two revised patient measures of safety that are practical, feasible and simple for patients to complete, responding directly to the need to enhance patient acceptability of the original measure.[19, 20] PMOS-30 included less negatively worded items compared to PMOS-44 (37% as opposed to 57%), and the internal reliability of PMOS-30 was established. Reducing the number of negatively worded items addressed the potential for unreliable responses, as previous research suggests that reverse worded items can be contaminated by inattention and confusion.[33] We retained a minimum of two items per domain across the nine domains known to contribute to safety incidents in hospitals and which formed the basis of the original measure[13, 14] informed by the YCFF.[15] In addition, and in collaboration with our patient representatives, items were rephrased to facilitate greater patient understanding.. One domain demonstrated an acceptable Cronbach's alpha coefficient and acceptable average inter-item correlation: Communication and teamworking. Four domains did not demonstrate an acceptable Cronbach's alpha coefficient but demonstrated an acceptable average inter-item correlation: Organisation and care planning, Access to resources, Ward type and layout, and Staff roles and responsibilities. Three domains (two item domains) demonstrated acceptable inter-item correlations: Information flow, Staff training, and Delays. The reduction in the number of items may have resulted in lowering the reliability statistics of the domains, however this is to be expected as a reliability coefficient generally increases as the number of items increases.[29] Interestingly the domains which performed poorest (e.g. Ward type and layout; Access to resources) also performed poorest in the original PMOS validation study[14]. Collectively, these findings demonstrate the stability of the original domains over time, even within a shorter, revised

For peer review only - http://bmjopen.bmj.com/site/about/guidelines.xhtml

measure and suggests that the conceptual underpinnings and psychometric properties of the original measure have been preserved.

The 10 most highly correlated items that were included in both PMOS-44 (original measure) and PMOS-30 formed the briefer standalone measure (PMOS-10), which was ratified in collaboration with patient representatives to establish face validity. A good alpha coefficient demonstrated the internal reliability of PMOS-10, and convergent validity was established. The strength of the correlation (moderate) suggests that although PMOS-10 ‘converges’ with another known measure of care experience from the patient perspective (FFT), it is indeed measuring something different. To determine disciminant validity, we examined whether PMOS-10 was able to discriminate meaningfully at the ward level, with a view to PMOS-10 being used as a tool to identify those wards which might benefit most from further data gathering efforts (i.e PMOS-30) and improvement as part of the PRASE intervention. There was no significant main effect of ward and no significant differences between wards. Therefore, based on our anlayses of nine wards, we cannot purport that PMOS-10 is able to meaningfully discrimiatate between wards.

There has been substantial research and policy focus on patient involvement in patient safety.[34-37] Patients providing feedback about the safety of their care is one way for patients to be enaged in patient safety.[38] The collection of feedback on patient satisfaction and experiences of care are also commonplace.[4] In terms of safety specifically, there is a wealth of research in support of patients providing feedback about the safety of their care,[8,9] and the PRASE intervention, is the first of its kind to collect patient feedback about safety on an on-going basis to support service improvement. Vincent and colleagues’[3] emphasised that patients and carers play an essential role in safety monitoring, but are often an underused resource. To move from patients and carers being an underused resource in the monitoring of safety, we need to ensure that the measures we do have to capture their perspective are acceptable to patients and carers, are not burdensome to complete and are reliable and valid. The research presented in this paper responds directly to these needs.

Strengths and limitations

A strength of the development of PMOS-30 is the involvement of six researchers/research nurses with an extensive amount of experience of facilitating PMOS (original measure) data collection, and the involvement of two hospital volunteers and two patient representatives. This provided a rich source of data regarding potential rephrasing of items; leading to considerable face validity. A further strength is that the reliability testing of PMOS-30 took

For peer review only - http://bmjopen.bmj.com/site/about/guidelines.xhtml

place in a variety of wards across three hospital trusts. The involvement of two patient represenatives in the development of PMOS-10 is a key strength.

A limitation of our work is that our studies only included patient participants who could understand English. Therefore, further testing would be required if PMOS-30 and PMOS-10 were to be translated into other languages. For PMOS-30, some of the domains failing to achieve the Cronbach's alpha recommendation of .70 is a limitation, even though the average inter-item and inter-item correlations were within the optimum range. For PMOS-10, although the wards recruited were from a variety of medical and surgical specialities, a limitation is the involvement of only one hospital trust. A further limitation is that disciminant validity was not established for PMOS-10. Finally, PMOS-30 had a high amount amount of missing data which were mainly not applicable responses, therefore it is possible that a listwise deletion approach may have implcations for generalisation.

Implications for health services researcher, policy and practice

By futher refining an existing measure, the findings respond to previous research which called for the acceptability of PMOS to be enhanced. The length and some content of the original measure were problematic, and posed issues relating to participant fatigue and burden. The output of a highly reliable and valid shortened diagnostic version of PMOS, which retains the psychometric properties and conceptual underpinnings of the original measure, is integral to the wider adoption and ongoing implementation of the PRASE intervention. The high recruitment response rate (92%) for PMOS-10 strengthens the argument that the measure is acceptable to patients, and these findings position PMOS-10 as a reliable and valid brief standalone measure for on-going monitoring of ward safety performance from the patient perspective. To further validate PMOS-10, research is needed to assess the discriminant validity PMOS-10 on larger scale, in addition to further exploring convergent validity by examining associations with other indicators of patient safety.

Conclusion

Two revised, shortened versions of the original PMOS-44 (PMOS-30 and PMOS-10) were produced to capture patient feedback about safety in hospital, and these measures demonstrated acceptable reliability and validity. PMOS-30 was produced to serve a diagnostic function to be used, alongside the PRASE intervention as a whole. PMOS-10 was developed to be used as a brief standalone measure for on-going monitoring of ward safety performance.

For peer review only - http://bmjopen.bmj.com/site/about/guidelines.xhtml

Twiter: @Gemma_Louch

Competing interests: None declared.

Acknowledgements: We would like to thank the Yorkshire Quality and Safety Research (YQSR) Group patient panel for reviewing the PMOS-10 measure, and we would like to thank the two members of the panel and two hospital volunteers for their time and input into revising the PMOS-30 measure. We would also like to thank the patient participants and the staff on the wards for their contribution to the research.

Funding statement: The research was supported by The Health Foundation, the National Institute for Health Research (NIHR) Yorkshire and Humber Patient Safety Translational Research Centre (NIHR YH PSTRC), and NIHR CLAHRC Yorkshire and Humber www.clahrc-yh.nihr.ac.uk. The PMOS-30 and PMOS-10 shortening exercises utilised data from a wider study funded by the NIHR under its programme Grants for Applied Research scheme ('Improving patient safety through the involvement of patients', RP-PG-0108-10049). The views expressed in this article are those of the author(s) and not necessarily those of The Health Foundation, the NHS, the NIHR, or the Department of Health and Social Care.

Author contribution: GL led on the development of PMOS-30, carried out the related analyses and drafted the manuscript. JH carried out the PMOS-10 shortening statistical analyses. CR led on the PMOS-10 data collection, and GL, AA and CR carried out the related analyses. SM and CM supported the studies at all stages, in particular the patient and public involvement and engagement element of this work. RL provided guidance and input at all stages. All authors provided comments and approved the final version.

Ethics approval: Ethical approvals were obtained from the University of Bradford, Humanities, Social and Health Sciences Research Ethics Panel, and from the East Midlands - Leicester Central Research Ethics Committee.

Data sharing statement: The data that support the findings of this study are available from the corresponding author, GL, upon reasonable request. PMOS-44, PMOS-30, PMOS-10 and 'easy read' versions which have subsequently been produced can be requested via https://www.improvementacademy.org/tools-and-resources/patient-reporting-and-action-for-a-safe-environment.html (freely available).

For peer review only

For peer review only - http://bmjopen.bmj.com/site/about/guidelines.xhtml

References

1 Pronovost PJ, Goeschel CA, Marsteller JA, Sexton JB, Pham JC, Berenholtz SM. Framework for patient safety research and improvement. Circulation. 2009 Jan 20;119(2):330-7.

2 Pronovost PJ, Wachter RM. Progress in patient safety: a glass fuller than it seems. American Journal of Medical Quality. 2014 Mar;29(2):165-9.

3 Vincent C, Burnett S, Carthey J. The measurement and monitoring of safety: drawing together academic evidence and practical experience to produce a framework for safety measurement and monitoring. The Health Foundation; 2013.

4 Coulter A, Fitzpatrick R, Cornwell J. Measures of patients' experience in hospital: purpose, methods and uses. London: King's Fund; 2009 Jul.

5 Francis R. Report of the Mid Staffordshire NHS Foundation Trust public inquiry: executive summary. The Stationery Office; 2013 Feb 6.

6 Keogh B. Review into the quality of care and treatment provided by 14 hospital trusts in England: overview report. 2013.

7 National Advisory Group on the Safety of Patients in England. A Promise to Learn a Commitment to Act: Improving the Safety of Patients in England. National Advisory Group on the safety of patients in England; 2013.

8 O'Hara JK, Armitage G, Reynolds C, Coulson C, Thorp L, Din I, Watt I, Wright J. How might health services capture patient-reported safety concerns in a hospital setting? An exploratory pilot study of three mechanisms. BMJ Qual Saf. 2016 Feb 4:bmjqs-2015.

9 Ward JK, Armitage G. Can patients report patient safety incidents in a hospital setting? A systematic review. BMJ Qual Saf. 2012 Aug 1;21(8):685-99.Quality & Safety. 2012;21(8):685–99.

10 Armitage G, Moore S, Reynolds C, Laloë PA, Coulson C, McEachan R, Lawton R, Watt I, Wright J, O'Hara J. Patient-reported safety incidents as a new source of patient safety data: an exploratory comparative study in an acute hospital in England. Journal of health services research & policy. 2018 Jan;23(1):36-43.

11 Lawton R, O'Hara J, Sheard L, Armitage G, Cocks K, Buckley H. Corbacho B, Reynolds C, Marsh C, Moore S, Watt I. Can patient involvement improve patient safety? A cluster randomized control trial of the Patient Reporting and Action for a Safe Environment (PRASE) Intervention. BMJ Qual Saf. 2017 Aug 1;26(8):622-31.

12 O'Hara JK, Lawton RJ, Armitage G, et al. The patient reporting and action for a safe environment (PRASE) intervention: a feasibility study. BMC Health Serv Res. 2016;16:676

For peer review only - http://bmjopen.bmj.com/site/about/guidelines.xhtml

13 Giles SJ, Lawton RJ, Din I, McEachan RR. Developing a patient measure of safety (PMOS). BMJ Qual Saf. 2013 Jul 1;22(7):554-62.

14 McEachan RRC, Lawton RJ, O'Hara JK, et al. Developing a reliable and valid patient measure of safety in hospitals (PMOS): a validation study BMJ Qual Saf 2014;23:565-573.

15 Lawton, R., et al., Development of an evidence-based framework of factors contributing to patient safety incidents in hospital settings: a systematic review. BMJ quality & safety, 2012:p. bmjqs-2011-000443.

16 Rolstad S, Adler J, Rydén A. Response burden and questionnaire length: is shorter better? A review and meta-analysis. Value in Health. 2011 Dec 1;14(8):1101-8.

17 Kelly L, Sizmur S, Käsbauer S, King J, Cooper R, Jenkinson C, Graham C. The Relational Aspects of Care Questionnaire: item reduction and scoring using inpatient and accident and emergency data in England. Patient related outcome measures. 2018;9:173.

18 Nielsen MB, Hystad SW, Eid J. The Brief Norwegian Safety Climate Inventory (Brief NORSCI)–Psychometric properties and relationships with shift work, sleep, and health. Safety science. 2016 Mar 1;83:23-30.

19 Louch G, O'hara J, Mohammed MA. A qualitative formative evaluation of a patient-centred patient safety intervention delivered in collaboration with hospital volunteers. Health Expectations. 2017 Oct;20(5):1143-53.

20 Taylor N, Hogden E, Clay-Williams R, Li Z, Lawton R, Braithwaite J. Older, vulnerable patient view: a pilot and feasibility study of the patient measure of safety (PMOS) with patients in Australia. BMJ open. 2016 Jun 1;6(6):e011069.

21 Louch G, Mohammed MA, Hughes L, O'hara J. "Change is what can actually make the tough times better": A patient-centred patient safety intervention delivered in collaboration with hospital volunteers. Health Expectations. 2019 Feb;22(1):102-13.

22 Department of Health. The NHS Friends and Family Test Implementation Guidance. https://www.gov.uk/government/uploads/system/uploads/attachment_data/file/213047/NHS-Friends-and-Family-Test-Implementation-Guidance-v2.pdf (accessed April 2019).

23 IBM Corp. IBM SPSS statistics: Version 23. 0. 2016.

24 Hatcher, L. (1994). A Step-by-Step Approach to Using the SAS® System for Factor Analysis and Structural Equation Modeling. Cary, N.C.: SAS Instittute, Inc.

25 Kyriazos TA. Applied psychometrics: Sample size and sample power considerations in factor analysis (EFA, CFA) and SEM in general. Psychology. 2018 Aug 2;9(08):2207.

26 Guest G, Namey E. Public health research methods. Thousand Oaks, CA: Sage Publications; 2014. p.392.

For peer review only - http://bmjopen.bmj.com/site/about/guidelines.xhtml

27 Briggs SR, Cheek JM. The role of factor analysis in the development and evaluation of personality scales. Journal of personality. 1986 Mar;54(1):106-48.

28 Bland JM, Altman DG. Statistics notes: Cronbach's alpha. BMJ. 1997 Feb 22;314(7080):572.

29 Tavakol M, Dennick R. Making sense of Cronbach's alpha. International Journal of Medical Education. 2011;2:53.

30 Clark LA, Watson D. Constructing validity: Basic issues in objective scale development. Psychological assessment. 1995 Sep;7(3):309.

31 Marteau TM, Bekker H. The development of a six-item short-form of the state scale of the Spielberger State—Trait Anxiety Inventory (STAI). British Journal of Clinical Psychology. 1992 Sep 1;31(3):301-6.

32 Cohen J. A power primer. Psychological bulletin. 1992 Jul;112(1):155.

33 Van Sonderen E, Sanderman R, Coyne JC. Ineffectiveness of reverse wording of questionnaire items: Let's learn from cows in the rain. PloS one. 2013 Jul 31;8(7):e68967.

34 Davis RE, Sevdalis N, Vincent CA. Patient involvement in patient safety: How willing are patients to participate?. BMJ Quality & Safety. 2011 Jan 1;20(1):108-14.

35 Vincent CA, Coulter A. Patient safety: what about the patient?. BMJ Quality & Safety. 2002 Mar 1;11(1):76-80.

36 O'Hara J, Lawton R. At a crossroads? Key challenges and future opportunities for patient involvement in safety. BMJ Quality & Safety. 2016;0:1-4. doi:10.1136/bmjqs-2016-005476

37 World Health Organization. Patients for Patient Safety Partnerships for Safer Health Care. Geneva: World Health Organization WHO. 2013. Available from: http://www.who.int/patientsafety/patients_for_patient/PFPS_brochure_2013.pdf

38 The Health Foundation. Evidence scan: Involving patients in improving safety. London: The Health Foundation. 2013.

For peer review only - http://bmjopen.bmj.com/site/about/guidelines.xhtml

COSMIN Study Design checklist for Patient-reported outcome measurement instruments*

**General recommendations for the design of a study on measurement properties**	Y/N
Research aim	
1 Provide a clear research aim, including (1) the name and version of the PROM, (2) the target population, and (3) the measurement properties of interest	Y
PROM	
2 Provide a clear description of the construct to be measured	Y
3 Provide a clear description of the development process of the PROM, including a description of the target population for which the PROM was developed	Y and in original validation study.
4 The origin of the construct should be clear: provide a theory, conceptual framework (i.e. reflective or formative model) or disease model used or clear rationale to define the construct to be measured	Y and in original validation study.
5 Provide a clear description of the structure of the PROM (i.e. the number of items and subscales included in the PROM, instructions given and response options) and its scoring algorithm	Y
6 Provide a clear description of existing evidence on the quality of the PROM	Y
7 Provide a clear description of the context of use*	Y
Target population	
8 Provide a clear description of in- and exclusion criteria to select patients, e.g. in terms of disease condition and characteristics like age, gender, language or country, and setting (e.g. general population, primary care or hospital/rehabilitation care)	Y
9 Provide a clear description of the method used to select the patients for the study (e.g. convenience, consecutive, or random)	Y
10 Describe whether the selected sample is representing the target population in which the PROM will be used in terms of age, gender, important disease characteristics (e.g. severity, status, duration)	Y
**Content validity**	
Design requirements	
1 From the perspective of the patients: use an appropriate method for assessing (1) the relevance of each item for the patients' experience with the condition, AND (2) the comprehensiveness of the PROM, AND (3) the comprehensibility of the PROM instructions, items, response options, and recall period	Y
2 From the perspective of professionals: use	Covered in development of

For peer review only

an appropriate method for assessing<br>(1) the relevance of each item for the construct of interest, AND<br>(2) the comprehensiveness of the PROM	original measure.
3 Include professionals from all relevant disciplines	N/A
4 Evaluate each item in an appropriate number of patients or professionals<br>For qualitative studies<br>For quantitative (survey) studies	Y
5 Use skilled group moderators or interviewers	Y
6 Base the group meetings or interviews on an appropriate topic or interview guide	Y
7 Record and transcribe verbatim the group meetings or interviews	N
Analyses	
8 Use an appropriate approach to analyse the data	Y
9 Involve at least two researchers in the analysis	Y
**Internal consistency**	
**Design requirements**	
1 Check whether a scale or a subscale is unidimensional	Covered in original validation study.
2 Perform the analysis in a sample with an appropriate number of patients (taking into account expected number of missing values)	Y
3 Provide a clear description of how missing items will be handled	Y
Statistical methods	
4 For continuous scores: calculate Cronbach's alpha or Omega for each unidimensional scale or subscale	Y
5 For dichotomous scores: calculate Cronbach's alpha or KR-20 for each unidimensional scale or subscale	N/A
6 For IRT-based scores: calculate standard error of theta (SE (θ)) or reliability coefficient of estimated latent trait value (index of (subject or item) separation) for each unidimensional scale or subscale	N/A
**Hypotheses testing for construct validity**	
**A. Comparison with other outcome measurement instruments (convergent validity)**	
Design requirements	
1 Formulate hypotheses about expected relationships between the PROM under study and other outcome measurement instrument(s)	Y
2 Provide a clear description of the construct(s) measured by the comparator instrument(s)	Y
3 Use comparator instrument(s) with sufficient measurement properties	Y
4 Perform the analysis in a sample with an appropriate number of patients (taking into account expected number of missing values)	Y
5 Use an appropriate time schedule for assessments of the PROM of interest and comparison instruments	Y
Statistical methods	

For peer review only

6 Use statistical methods that are appropriate for the hypotheses to be tested	Y
7 Provide a clear description of how missing items will be handled	Y
**B. Comparison between subgroups (discriminative or known-groups validity)**	
Design requirements	
1 Formulate hypotheses regarding mean differences between subgroups	Y
2 Provide an adequate description of important characteristics of the subgroups, such as disease or demographic characteristics	Y
3 Perform the analysis in a sample with an appropriate number of patients (taking into account expected number of missing values)	Y
Statistical methods	
4 Use statistical methods that are appropriate for the hypotheses to be tested	Y
5 Provide a clear description of how missing items will be handled	Y

*Only selected components are included relevant to the study.

For peer review only
